# Spatiotemporal Response and Phytopotential of *Typha domingensis* for Management of Aquatic Metal Pollution on the Central African Copperbelt

**DOI:** 10.1002/ece3.71039

**Published:** 2025-02-28

**Authors:** Kennedy O. Ouma, Agabu Shane, Concillia Monde, Stephen Syampungani

**Affiliations:** ^1^ Department of Zoology and Aquatic Sciences, School of Natural Resources Copperbelt University Kitwe Zambia; ^2^ Copperbelt University Africa Centre of Excellence for Sustainable Mining (CBU‐ACESM) Kitwe Zambia; ^3^ Department of Environmental Engineering, School of Mines and Mineral Sciences Copperbelt University Kitwe Zambia; ^4^ Chair‐Environment & Development, Oliver R Tambo Africa Research Chairs Initiative (ORTARChI) Copperbelt University Kitwe Zambia; ^5^ Forest Science Postgraduate Program, Department of Plant and Soil Sciences, Plant Sciences Complex University of Pretoria Pretoria South Africa

**Keywords:** excluder, macrophyte, mining landscape, nature‐based management, phytoindicator, phytoremediation

## Abstract

Anthropogenically accelerated metal pollution of lotic systems draining mining landscapes remains a global concern due to its non‐degradability and ecotoxic nature. Nevertheless, the efficacy of macrophytes as indicators and remediators of metal(loid) pollutants in aquatic ecosystems in mining regions is recognised globally. However, in the mineral‐rich Central African Copperbelt (CACB), there is limited research on the effectiveness of macrophytes for mining pollution management. Therefore, this study investigated the phytopotential of *Typha domingensis* Pers. (Typhaceae), as a nature‐based approach for managing aquatic metal(loid) pollution on the CACB. A total of 252 samples each for sediment and *T. domingensis* were collected monthly between May 2022 and April 2023 from seven streams in the Kansanshi sub‐catchment of the CACB and analysed to evaluate the spatiotemporal variability of the phytoindication and phytoremediation potential of *T. domingensis* for As, Cu, Pb and Zn in stream sediments. Metal(loid)s in *T. domingensis* at 71% of the streams were predominantly accumulated in the below‐ground biomass with mean concentrations of 3–6 mg/kg As, 9–259 mg/kg Cu, 25–36 mg/kg Pb and 38–69 mg/kg Zn. The BAF was above 1 for As (0.8–2.6), Pb (10.7–24.3) and Zn (1.4–4.3), indicating *T. domingensis* as an accumulator of these elements but an excluder for Cu (BAF < 1). Additionally, TF was above 1 for As (1.5–2.0), Pb (1.4–1.5) and Zn (1.0–2.0), indicating the macrophyte's extraction efficacy. For Cu (TF < 1), the limited translocation characterised *T. domingensis* as a potential metal phytostabiliser. Similar seasonal trends for BAF and TF for As, Cu, Pb and Zn were also observed. Therefore, the spatiotemporal response of *T. domingensis* as a phytoindicator and phytoremediator of metal contaminants in sediments should be considered in the nature‐based management of CACB's aquatic ecosystems.

## Graphical abstract

The study provides an eco‐friendly, nature‐based approach for biomonitoring and management of mining pollution to ensure aquatic ecosystem sustainability in sub‐Saharan Africa mining landscapes. We investigated the phytoindication and phytoremediation potential of *Typha domingensis* for the management of mining pollution in tropical lotic ecosystems. The bioaccumulation factor (BAF) and translocation factor (TF) of *T. domingensis* for As, Cu, Pb and Zn contamination in streambed sediments illustrate the potential dual role of *T. domingensis* in mining pollution management.

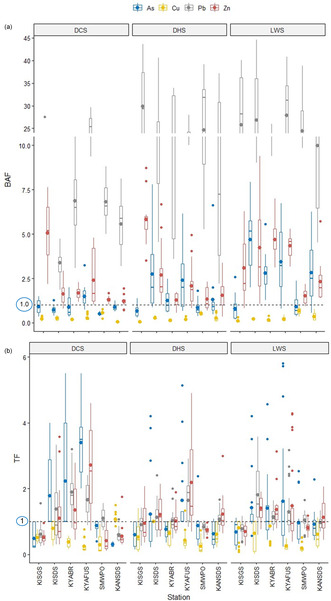

## 1 Introduction

Anthropogenically accelerated aquatic metal pollution in lotic systems draining mining landscapes remains a global concern due to the non‐degradability and ecotoxic nature of metal(loid)s (Luckeneder et al. 2021; Niu et al. 2021; Zhou et al. 2020). Industrial, artisanal and small‐scale mining are among the significant contributors of metal contaminants to lotic ecosystems worldwide including sub‐Saharan Africa (Briffa et al. 2020; Schwartz et al. 2021). In sub‐Saharan Africa, the mineral‐rich Central African Copperbelt (CACB), characterised by the Katanga Supergroups metasedimentary rocks, stretches from the Congolese Copperbelt (CCB), Katanga Province of the Democratic Republic of Congo (DRC) into the Zambian Copperbelt (ZCB) covering the North‐Western and Copperbelt Provinces of Zambia (Hitzman et al. 2012). The CACB extends 700 km in the arcuate fold‐thrust Lufilian arc, ‘a Pan‐African orogenic belt enriched with Cu‐Co deposits’ (Rainaud et al. 2005). The environmental and health impacts of mining in the CACB are widely documented (Albanese et al. 2014; Calderon et al. 2021; Kříbek et al. 2023; M'kandawire et al. 2017; Muimba‐Kankolongo et al. 2022; Mwaanga et al. 2019). The environmental pollution from mining on the CACB impedes the provision of aquatic ecosystem goods and services besides the negative impacts on human and animal health (Peša 2021, 2022). The rich networks of aquatic ecosystems on the CACB form conveyance routes and sinks for mining wastes from terrestrial and atmospheric environments and are, therefore, more susceptible to mining pollution (Kříbek et al. 2023; Muimba‐Kankolongo et al. 2022).

The intensification of industrial and artisanal mining on the CACB has accelerated the release of significant amounts of metals into the environment, which eventually contaminate its lotic ecosystems (Garrett 2000; Vareda et al. 2019; Yabe et al. 2010). For instance, in the Katanga Province of the DRC, Atibu et al. (2013, 2016) reported elevated concentrations of Cu (370–47,468 mg/kg) and Co (240–13,199 mg/kg) in sediments of Luilu and Musonoje rivers. The authors also observed extremely high Co (2046 mg/kg), Cu (24,098 mg/kg), Zn (5463 mg/kg), and Pb (3340 mg/kg) content in the sediments of Lubumbashi River, triggered by an inflow of effluents from mining and industrial activities. In the Congo River, the high Cr (117 mg/kg), Ni (8815 mg/kg), Zn (88 mg/kg) and Pb (15 mg/kg) levels in sediments were associated with anthropogenic pollution sources (Mwanamoki et al. 2015). In the same Congo River, Mata et al. (2020) correlated ‘heavy‐to‐extreme’ sediment enrichment with Co (37.1 mg/kg), Ni (139.9 mg/kg), Cu (281.5 mg/kg) and Pb (2009 mg/kg). In Atibu et al. (2018) findings, extreme sediment enrichment with Cu (176,801 mg/kg), Co (18,434 mg/kg) and Pb (899 mg/kg), and in macrophyte biomass with Cu (34,061 mg/kg) and Co (5050 mg/kg) and Pb (230 mg/kg) in DRC's Dilala, Luilu and Mpingiri rivers emphasised the anthropogenic contribution to aquatic pollution on the CCB. Furthermore, Banze wa Mutombo et al. (2022) recently investigated the ecological impact of Cu–Co–Au mining on the sediment quality of the Mira and Kimpulande rivers draining the Haut‐Katanga mining region. High contents of Co (2760 mg/kg), Zn (282.2 mg/kg) and As (69.2 mg/kg) were found in the Kimpulande River. In Mira, sediments were extremely enriched with Cu (9915 mg/kg), Ni (98.1 mg/kg), Cr (81.3 mg/kg) and Pb (86.1 mg/kg). Several other studies on the CCB have shown similar sediment pollution trends and associated aquatic ecological risks (Bamba Bukengu et al. 2017; De Putter et al. 2011; Mees et al. 2013; Sakib 2023).

On the ZCB, several studies have monitored aquatic metal pollution, particularly in the Kafue river basin draining the North‐Western and Copperbelt provinces. In the Kafue River, for instance, Choongo et al. (2005), M'kandawire et al. (2017) and Ikenaka et al. (2010) noted the anthropogenic sediment enrichment with Cu (15–12,906 mg/kg), Co (6–748 mg/kg), Mn (197 mg/kg) and As (6–52 mg/kg) with likely adverse biological effects on aquatic biota. Kříbek et al. (2023) also reported elevated Cu (7006 mg/kg) and As (655 mg/kg) in the Changa River, Chingola. In the same study, the Uchi River in Kitwe had the highest Co (1222 mg/kg) and Pb (121 mg/kg) concentrations, mainly from industrial effluents. In addition, Ntengwe and Maseka (2006) observed Zn (0.18 mg/kg) and Ni (1.86 mg/kg) enrichment in the sediments of two Kafue River tributaries, the Chambishi and Mwambashi streams in the Copperbelt Province. In north‐western Zambia, Hasimuna et al. (2022), Hasimuna et al. (2021) reported sediment contamination by Cu (10 mg/kg) and Fe (26 mg/kg); and Cu (0.04 mg/kg), Fe (0.47 mg/kg) and Zn (0.4 mg/kg) in large‐scale yellowfish ( *Labeobarbus marequensis* ) from Solwezi and Kifubwa rivers, from mining activities in Solwezi District.

In light of the above case studies, there is an urgent need to explore appropriate interventions for mining pollution to ensure the sustainable provision of aquatic ecosystem goods and services in the region. Consequently, the use of macrophytes is one viable complementary and eco‐friendly nature‐based approach for managing aquatic metal pollution across the CACB (Calderon et al. 2021; Odoh et al. 2019; Sytar et al. 2016). Macrophytes employ various phytoremediation mechanisms, including phytovolatilisation, phytoextraction, phytostabilisation, phytodegradation, phytostimulation, rhizodegradation and rhizofiltration (Kafle et al. 2022; Simmer and Schnoor 2022). Submerged, free‐floating and emergent macrophytes are excellent phytoindicators and phytoremediators of aquatic metal pollution in engineered or natural wetlands (Eid, Galal, Sewelam, et al. 2020; Eid, Galal, Shaltout, et al. 2020; Heisi et al. 2023). Several emergent macrophyte genera, including *Typha*, *Phragmites*, *Cyperus*, *Echinochloa* and *Scirpus*, are indicators and potential remediators of metals in aquatic environments (Bonanno 2013; Futughe et al. 2020). For instance, Bonanno et al. (2017) reported metal accumulations up to 1890 mg/kg Al, 2.86 mg/kg As, 18.5 mg/kg Cr, 13.7 mg/kg Pb and 122 mg/kg Zn in a *Typha* spp. dominated natural wetland in Sicily, Italy, which is indicative of a high phytoremediation capacity. The phytostabilisation ability of *Phragmites australis* and *T. latifolia* for Fe, Cu, Zn, Pb and Ni in riverine wetlands receiving mining effluents has also been demonstrated (Milke et al. 2020; Nabuyanda et al. 2022). In a study of metal phytoremediation by 18 macrophyte species in Catania, Italy, by Bonanno et al. (2018), *Typha domingensis* exhibited excellent phytostabilisation capacity (TF > 1) for As (0.27), Cr (0.47), Cu (0.73), Ni (0.63), Pb (0.18) and Zn (0.87).

In the CACB, approximately 32% (360,000 km^2^) of the CCB and 5% (37,649 km^2^) of the ZCB are wetlands (Bwangoy et al. 2010; Taylor et al. 1995). Most of these extensive minetrophic riverine wetlands are dominated by monospecific stands of *Typha domingensis* Pers. (Typhaceae). Commonly known as the southern cattail, *T. domingensis* is a highly invasive and ecologically relevant helophyte native to the tropics and subtropics (Sesin et al. 2021; WCSPF 2023). *T. domingensis* provides various ecosystem functions and benefits, including flood regulation, bank and sediment stability, biodiversity and habitat conservation, water purification, and contaminant retention or immobilisation (Bowden et al. 2007). With 10–13 congenerics, *Typha* spp. are also socioecologically and economically important to local communities as food, medicine, and biomass sources for crafts and other uses (Akkol et al. 2011; Bussmann et al. 2021). *T. domingensis* is a perennial, rhizomatous helophyte that grows rapidly (1.5–5 m tall), has a high reproduction rate and dominates even under stressful conditions due to its high morphological and functional plasticity (Bussmann et al. 2021; de Oliveira et al. 2022; Minkina, Fedorenko, Nevidomskaya, Konstantinova, et al. 2021).

The bioindication and phytoremediation ability of *T. domingensis* for metal(loid) pollutants in aquatic systems globally has been extensively documented (Al‐Sodany et al. 2021; Hadad et al. 2018; Sesin et al. 2021). High metal accumulation has been reported for *T. domingensis* with bioaccumulation factors between 7 and 3618 for several elements, including As, Co, Cu, Ni, Pb, Zn and Hg (Compaore et al. 2020; Lominchar et al. 2015). *T. domingensis* is also a suitable stabiliser for Cr, Ni, Zn, Fe, Cd and Cr in impacted aquatic environments (Dube et al. 2019; Hadad et al. 2018). Studies on the performance of *T. domingensis* for aquatic metal remediation in Europe (Basallote et al. 2023), Australia (Adams et al. 2013), Latin America (Gómez‐Bernal et al. 2017), Asia (Subramanian et al. 2022), the Middle East (Soudani et al. 2022) and North Africa (Abdelaal et al. 2021; Hamad 2023) have yielded promising results.

However, the influence of local geochemistry, hydrology and mineralogical peculiarities across the CACB on the phytoindication and phytoremediation performance of *T. domingensis* needs consideration. Besides site‐specific variability in stream limnochemistry, hydroperiodicity also plays a significant role in determining metal fluxes in stream sediments (Byrne et al. 2020). Seasonal variability in hydrological characteristics, such as streamwater velocity and discharge, can influence terrestrial inputs, sediment erosion, transport and deposition in streams (Lintern et al. 2018). These processes, in turn, affect the distribution and concentration of metals in sediments. For instance, episodic flooding events increase overland soil erosion, re‐suspension and sediment transport, leading to higher metal concentrations in downstream sediments (Ponting et al. 2021; Yao et al. 2022). Similarly, increased sediment deposition during low flows can result in localised elevated metal concentrations in streams. The timing and intensity of rainfall events and anthropogenic activities in mining landscapes can further modulate stream hydrogeochemistry and metal fluxes in stream sediments (Dendievel et al. 2022; Xue et al. 2022). Additionally, surface and groundwater interaction can exhibit significant influence on seasonal metal fluxes in stream sediments (Wang et al. 2023; Xiao et al. 2023). Hence, establishing the spatial and seasonal stream health condition via sediment quality assessment using established geochemical indices, such as enrichment factor (EF), metal pollution index (MPI), pollution load index (PLI), toxicity response index (TRI), probable effect quotient (PEC) and (Enuneku et al. 2018; Sojka and Jaskuła 2022); and the biological condition through biotic indices, such as bioaccumulation factor (BAF), translocation factor (TF) and metal accumulation index (MAI) (Ariyachandra et al. 2023; Kutty and Al‐Mahaqeri 2016) would provide an overall indication of the ecological condition of aquatic ecosystems in mining impacted regions.

Nevertheless, selected studies outside the CACB have documented *T. domingensis* as both an extractor and stabiliser of metals depending on the predominant region‐specific mineralogical and geochemical attributes (Abdelaal et al. 2021; Al‐Sodany et al. 2021; Bonanno et al. 2017). Considering the CACB's mineralogical diversity, peculiarity and considerably low advancement in mining waste management technologies, aquatic systems are vulnerable to natural and anthropogenic metal pollution (Hitzman et al. 2012; Kříbek et al. 2023). Given the recent expansion of industrial, artisanal and small‐scale mining in the CACB, terrestrial and aquatic metal pollution is projected to increase in the next decade (Besa 2023; Peša 2022; ZCM 2018). As previously highlighted in this section, the cascading environmental impacts on the lotic ecosystems of the CACB, which warrant urgent interventions, cannot be overemphasised. Furthermore, the increasing magnitude of the aquatic ecological risks of mining pollution, ranging from water and sediment pollution, threats to biodiversity, and impairment of ecosystem services in its river basins, have been extensively documented (Kříbek et al. 2023; Ouma et al. 2022). Therefore, there is an urgent need to explore eco‐friendly complementary interventions to mitigate the impacts of mining on aquatic ecosystems in the region.

Phytoremediation using the native *T. domingensis* is one viable nature‐based eco‐friendly approach to mitigate the ecological impacts of mining on aquatic systems in the CACB (Calderon et al. 2021; Soudani et al. 2022). However, the promising metal phytoindication and phytoremediation potential of *T. domingensis* in the CACB have not been adequately demonstrated except for a few baseline studies (Kříbek et al. 2011; Nabuyanda et al. 2022). Furthermore, the spatiotemporal response and phytopotential of *T. domingensis* as a bioindicator for managing aquatic metal pollutants in the CACB remain understudied and hence not well established. This scientific knowledge and understanding will be useful in designing region‐specific nature‐based interventions for managing aquatic metal pollution across the uniquely mineral‐rich CACB. Therefore, this study aimed to determine the spatiotemporal response of *Typha domingensis* as (1) a potential phytoindicator of aquatic metal pollution in stream ecosystems of the CACB by detecting the presence and concentrations of metals in the sediment matrix; and (2) a potential phytoremediator through metal bioaccumulation or immobilisation mechanisms in sediments of the stream ecosystems draining the CACB. Furthermore, by incorporating the bioaccumulation and translocation efficiencies to characterise the efficacy of *T. domingensis* to phytostabilise or phytotransfer metals in stream sediments, the study provides scientific knowledge and insights into the double ecological potential of *T. domingensis* for nature‐based biomonitoring of aquatic metal pollution, and the eco‐friendly sustainable management of metal pollution of aquatic ecosystems in the CACB.

## 2 Materials and Methods

### 2.1 Study Area

**FIGURE 1:**
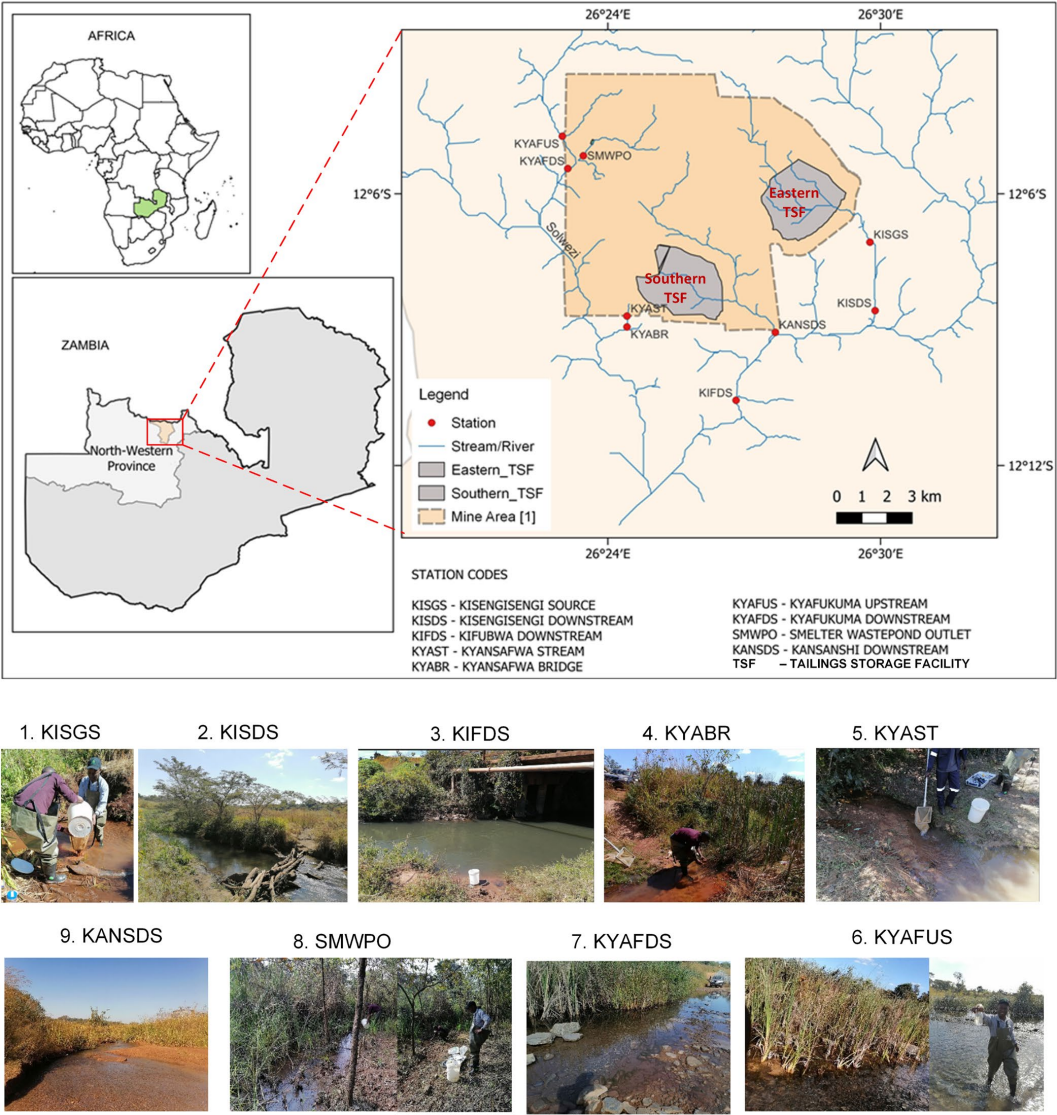
The geolocation and physical characteristics of the streams sampled in the Kansanshi sub‐catchment, north‐western Zambian Copperbelt. Inset plates 1–9 show the presence of extensive *Typha domingensis* –dominated riverine wetlands along the sampling sites.

The research was conducted in the north‐western ZCB of the CACB. Zambia's 125,826 km^2^ North‐Western Province (NWP), the ‘new’ Copperbelt (Figure 1), lies between 10°–18° S and 22°–33° E, at 872–1737 m above sea level (ZCM 2018; ZSA 2022). Angola borders the Province in the west, the Democratic Republic of Congo (DRC) in the north, while within the country, it is bordered by the Copperbelt Province south‐eastwards, the Central Province down south and the Eastern and Western Provinces in the northeast. The NWP altitude gently decreases from west to east from the DRC border and plateaus towards the Zambezi depression with intermittent uplifted surfaces. The Province lies in the tropical savanna, dominated mostly by humid subtropical with traces of tropical savanna and subtropical highland oceanic. Mean annual temperatures range between 18°C and 26°C. The annual trimodal seasonal pattern in NWP is marked by wet‐rainy, dry‐cold, and dry‐hot cycles from November to April, May to July, and August to October, respectively. The rainy season peaks in December–January at mean rainfall from 1200 to 1500 mm (CIAT‐World Bank 2017). The NWP mostly lies within Agro‐ecological Zone (AEZ) III (ca. 9,624,000 ha), while smaller proportions of miombo‐dominated forest cover interspersed with savanna grasslands extend to AEZ IIa (ca. 315,000 ha) and AEZ IIb (ca. 102,000 ha) (CIAT‐World Bank 2017; Tambo et al. 2021).

Three large copper mines, Kansanshi (Solwezi District), Lumwana (Mwinilunga District), and Kalumbila (Kalumbila District), operate within the NWP with a projected collective copper production of 640,000 of the 3 million metric tons Zambia's production target by 2030 (Besa 2023). A field study was conducted in the Kansanshi sub‐catchment (KSC), located between 26°20′–26°35′ E and 12°0′–12°12′ S within Solwezi District. The KSC drains a copper mining area in Solwezi District (Figure 1). Minerals in the KSC lie within the Katanga Supergroup sediments of the new ZCB. Copper primarily occurs on sulphide complexes (chalcopyrite and bornite) and mixed oxides (chrysocolla‐malachite), while gold deposits are associated with copper mineralisation (FQM 2020).

### 2.2 Study Sites and Sampling

Sampling was done from seven stream locations in the KSC: Kisengisengi source (KISGS), Kisengisengi downstream (KISDS), Kansanshi (KANSDS), Kyansafwa bridge (KYABR), Kyafukuma upstream (KYAFUS), Smelter Waste Pond outlet stream (SMWPO) and Kansanshi downstream (KANSDS) (Figure 1). Additionally, reference sediment samples (KSCRS‐01, KSCRS‐02 and KSCRS‐03) were collected from three sites along stream KSCRS in a protected non‐mining area within Solwezi District and composited for quality assurance and validation checks. The geolocation of each station was captured using a hand‐held (Garmin e‐trex 62 s) GPS. At all the stations, the extensive riverine wetland vegetation was predominantly dense monospecific stands of the native macrophyte *Typha domingensis* Pers. (Typhaceae) (Figure 1). Additional geophysical and hydrological characteristics of the stations are described in Table A1 in Appendix 1.

Samples were collected monthly between May 2022 and April 2023 to reflect Zambia's dry‐cold (DCS) (May–July), dry‐hot (DHS) (August–November) and long‐wet (LWS) (December–April) seasons. For quality assurance and quality control, triplicate 200 g sediment samples were collected using a precleaned plastic scoop at a depth of 0–5 cm at three points across the stream channel. The samples were stored in pre‐rinsed, labelled plastic bottles and transported at 4°C to ensure sample integrity before laboratory processing and analysis. Additionally, triplicate whole biomass samples of *T. domingensis* were manually uprooted from three randomised points in the riverine wetland at each site. The harvested samples were thoroughly washed in stream water and rinsed with distilled water to eliminate debris, sediment, periphyton and other foreign materials. The plant materials were placed in labelled polythene bags for further processing and laboratory analysis. A total of 252 samples were collected each for sediment and *T. domingensis* biomass.

### 2.3 Sample Preparation and Laboratory Analysis

Sediment samples were air‐dried, homogenised, and sieved through a series of 250‐ and 125‐μm pore‐size stainless steel sieves (BS 410, Endecotts Ltd., London, UK). The sieved samples were subsequently collected into 50‐g transparent zip‐lock polythene bags. The *T. domingensis* samples from each station were separated into above (shoot and leaves)‐ and below (rhizomes and roots)‐ground biomass, after which the young sprouts and senescing tissues were removed. Young plant parts are known to dedicate their energy to growth, while senescing plant parts primarily concentrate biometabolites rather than contaminants of interest (Munyai and Dalu 2023; Świerk and Szpakowska 2011). The sorted biomass was chopped into smaller pieces and air‐dried for 72 h, followed by oven‐drying at 70°C for 24 h. The dried samples were pulverised using a blender, homogenised, and passed through a series of 250‐ and 125‐μm mesh‐size plastic sieves into separate polythene bags for metal analysis.

The concentrations of As, Cu, Pb and Zn in the triplicate sediment and *Typha* samples per site per season were measured directly using a Niton portable‐XRF (pXRF) analyser (XL3t GOLDD+, Thermo Scientific, Waltham, MA, USA) fitted with a 50 kV anode X‐ray tube sample excitation system, an 80 MHz signal processor and a ‘Geometrically Optimised Large Area Drift Detector’ (GOLDD). X‐ray fluorescence (XRF) spectrometry is a rapid, high‐precision and non‐destructive analytical technique for determining multi‐elemental composition from environmental samples (Oyedotun 2018; Sirkovich et al. 2023). Before the analyses, the pXRF performs an internal calibration and only allows measurements upon verification of operational parameters. The GOLDD detects the intensities as counts, then internally converts the counts to elemental concentrations. The following instrument parameters were used: start‐up‐to‐warm‐up time 3–5 min; reading time 30 s; reading mode: all‐geo; unit ppm or %; error margin ± 2 SD; and limit of detection in mg/kg (As—4; Cu—10; Pb—5: Zn—7). The metal concentrations in the samples were read in six replicates at different surface points to obtain representative values. The pXRF readings were interpreted and displayed using the in‐built Thermo Scientific NDT software (ver. 8.5.1).

### 2.4 Data Analysis

All visualisations and statistical analyses (at *p* < 0.05) were performed in R version 4.3.2 (R Core Team 2023). Descriptive multivariate analyses of the spatiotemporal variability of metals in sediments and *T. domingensis* , sediment quality and *T. domingensis* phytopotential characteristics were conducted, and summarised in tables. Additionally, the distributions were visually presented in boxplots using the function ‘ggboxplot()’ in the ‘ggbupr()’ package (Kassambara 2023a). To examine the data quality, datasets were tested for the Shapiro–Wilk multivariate normality (Shapiro and Wilk 1965; Villasenor Alva and Estrada 2009) using the Shapiro–Wilk test function ‘mshapiro_test()’ in the ‘rstatix()’ library (Kassambara 2023b). Multivariate outlier tests were also conducted (*p* > 0.05) using the Mahalanobis distance function ‘mahalanobis_distance()’ in the ‘rstatix()’ library to flag extreme outliers. A two‐way factorial multivariate analysis of variance (MANOVA) using the function ‘manova()’ in the ‘stats()’ library (R Core Team 2023). The MANOVA test is based on the best linear combination of multiple dependent variables; hence, it reduces Type‐I error by maximising the discrimination between‐groups rather than within‐groups (Buttigieg and Ramette 2014). The two‐way MANOVA test was conducted to compare the spatiotemporal variability in (1) the concentrations of As, Cu, Pb, and Zn in sediments and *T. domingensis* ; and (2) bioaccumulation (BAF) and translocation factors (TF) of *T. domingensis* for As, Cu, Pb and Zn. Additionally, the partial Eta^2^ ($\eta_{p}^{2}$) statistic (Norouzian and Plonsky 2018) was used to measure the amount of variance accrued from the site, season and site–season interaction effects on the metal concentration in sediments and *Typha*, and the variability in BAF and TF for metals by *T. domingensis* (Richardson 2011).

To isolate the spatiotemporal variance of individual metals in sediments and *T. domingensis* , a Tukey HSD post hoc test using the R function ‘tukey_hsd()’ in the ‘rstatix()’ library (Kassambara 2023b) was further conducted on the joint significant variances following the two‐factor MANOVA tests. The Tukey HSD post hoc test output was visualised using the function ‘ggboxplot()’ embedded in the ‘ggpubr()’ package (Kassambara 2023a). Moreover, a linear discriminant analysis (LDA) post hoc test, implemented using the function ‘lda()’ in the ‘MASS()’ package (Venables and Ripley 2002), was conducted to jointly establish the significant variances from the two‐way MANOVA tests (Xanthopoulos et al. 2013). The LDA was used to determine specific site and seasonal variances and interaction effects of combined metal concentrations in stream sediments and *Typha* biomass. The LDA biplots were generated using ‘ggplot2()’ to visualise the site and season clusters for the joint effect of metals on sediment quality, concentrations in *T. domingensis* and the phytopotential of the macrophyte.

Stream sediment quality was evaluated based on the degree of enrichment and contamination with metals using the enrichment factor (EF) and metal pollution index (MPI) respectively (Elgendy et al. 2024). Additionally, the potential toxicity of metals in sediment was estimated from the toxicity response index (TRI) and mean probable effect level (m‐PEL). The metal accumulation index (MAI) for *T. domingensis* was also established as an indicator of its overall phytopotential (Liu et al. 2007). Table A2 in Appendix 1 details the input parameters, mathematical computation and interpretation of EF, MPI, TRI, m‐PEL and MAI outputs. The threshold values for EF = 1, MPI = 1, TRI = 5 and mPEL = 0.1 were considered as indicators of sediment enrichment or contamination (Prasad Ahirvar et al. 2023).

Furthermore, the natural ability of *T. domingensis* to phytoremediate metal‐contaminated stream sediments was estimated using the bioaccumulation factor (BAF) and translocation factor (TF). Plants with BAF > 1 are considered accumulators, while plants with TF < 1 and > 1 are considered stabilisers and extractors, respectively (Ariyachandra et al. 2023). The BAF is the ratio of the concentration of a metal in plant tissue relative to the concentration in the sediment, expressed as BAF = *M*_plant_/*M*_sediment_, where *M*_plant_ is the mean metal concentration (mg/kg) in *T. domingensis* , and *M*_sediment_ is the metal concentration (mg/kg) in the stream sediments (Ladislas et al. 2012). The TF is the ratio of the concentration of a metal in the shoot to the concentration in the roots and is expressed as TF = *M*_shoot_/*M*_roots_, where *M*_shoot_ is the metal concentration (mg/kg) in the shoot, and *M*_roots_ is the concentration (mg/kg) of the metal in the roots (Kutty and Al‐Mahaqeri 2016).

## 3 Results

### 3.1 Quality Checks for Analytical Accuracy

From Table 1 results, the pXRF recoveries for metals analysed in the stream sediments were within the acceptable range, although As recovery (166.2%) was extremely high. Generally, recoveries approaching 100% indicate increasing instrument accuracy, although recoveries of 85%–115% are generally a considerable range for accuracy assessment (APHA et al. 2017).

**TABLE 1:** Instrument detection limits, sediment sample and reference material (KSCRS) overall mean concentrations and recoveries for the analysed metals.

Element	LOD (mg/kg)	Sample (mg/kg)	KSCRS (mg/kg)	% recovery
As	4	6.98	4.20	166.19
Cu	10	418.30	490.90	85.21
Pb	5	5.89	5.46	107.88
Zn	7	49.6	44.28	112.00

### 3.2 Metal Distribution in Stream Sediments and *Typha domingensis* Biomass

#### 3.2.1 As, Cu, Pb and Zn in Stream Sediments

**FIGURE 2:**
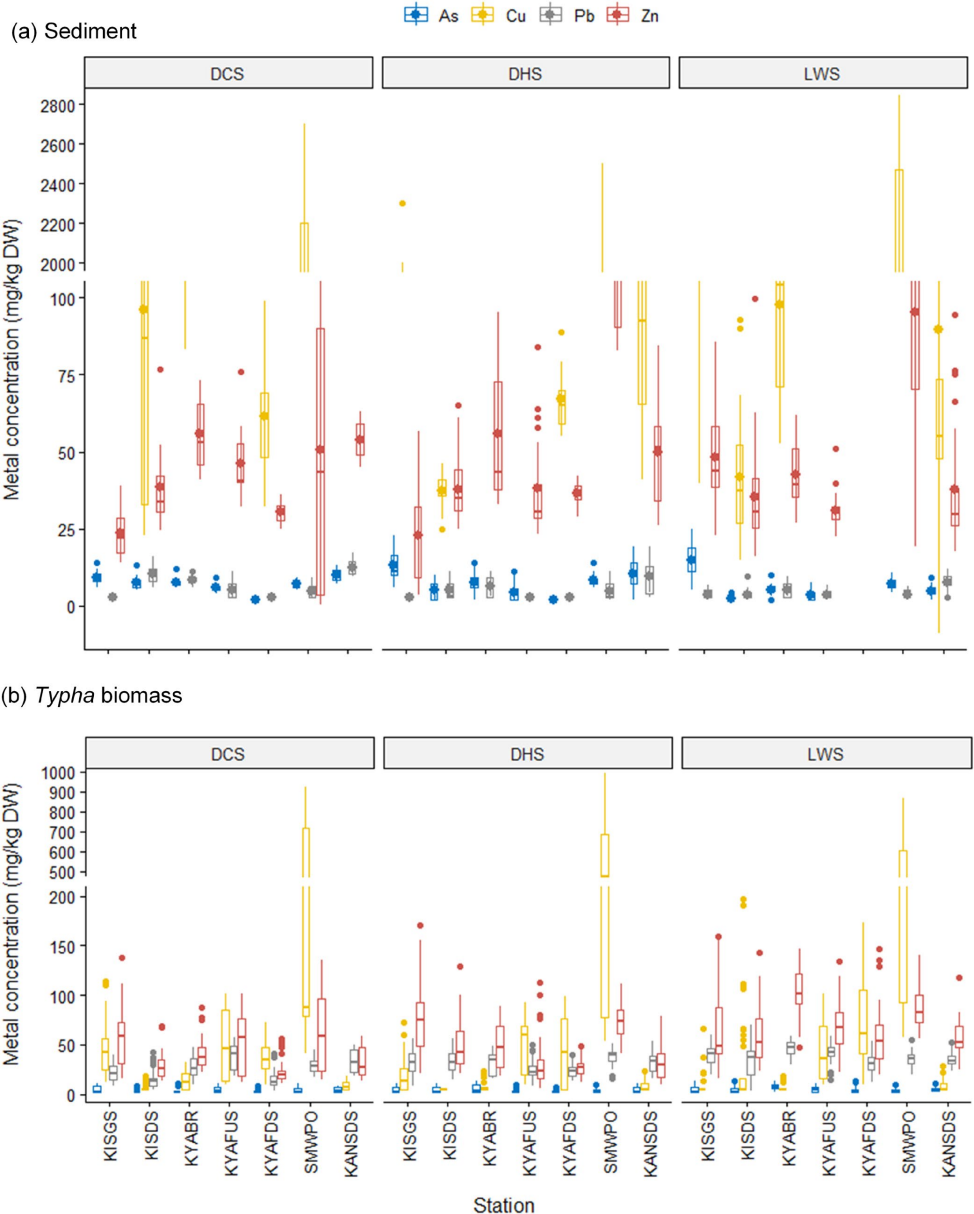
Multivariate boxplots illustrating the spatiotemporal variability of metal concentrations between May 2022 and April 2023 in (a) stream sediments and (b) *Typha domingensis* biomass from streams in KSC, northwestern Zambian Copperbelt.

The spatiotemporal distribution of As, Cu, Pb and Zn concentrations in stream sediments is shown in Figure 2a and Table A3 in Appendix 1. The lowest As concentration (2.3 mg/kg) was in KISDS during the LWS, while the highest concentration (14.7 mg/kg) was in KISGS also in the LWS. The mean Cu concentration ranged between 37 mg/kg at KISDS in the DHS and 1892 mg/kg at SMWPO in the DCS. The mean concentration of Pb (2.5 mg/kg) was lowest at KISGS and KYAFUS in the DCS and DHS, respectively. The highest Pb value (12.5 mg/kg) was reported at KANSDS during the DCS. Zn concentration (22.8 mg/kg) was lowest in the KISGS sediments but highest at SMWPO (126.1 mg/kg) during the DHS.

**FIGURE 3:**
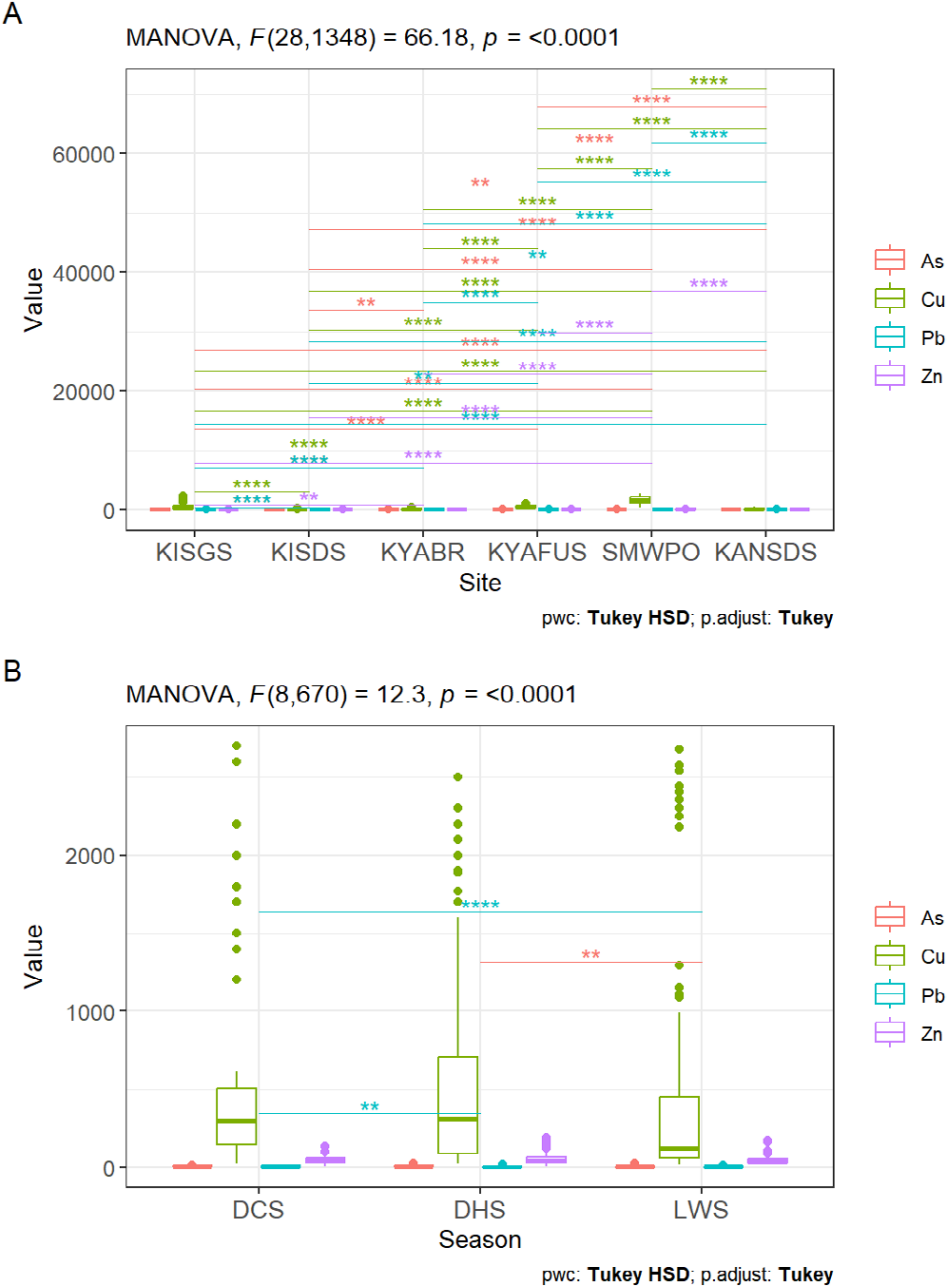
Tukey HSD post hoc tests for spatial (3A) and temporal differences (3B) in As, Cu, Pb and Zn in stream sediments in the KSC, north‐western Zambian Copperbelt. *Note:* *, **, *** and **** represent significant differences at *p* < 0.05, 0.01, 0.001 and 0.0001 respectively.

In Figure 3, Tukey HSD post hoc test was conducted for significant two‐way MANOVA on the joint site and seasonal variances of metal concentration in sediments (Section 3.5). Considering the pooled spatial metal distribution (Figure 3A), KYAFUS had a significantly lowest mean As concentration (4.2 mg/kg), while KISGS reported a significantly highest As (12.9 mg/kg) in the sediments (Tukey HSD, *p* < 0.0001). Cu was significantly lowest in KISDS (50.6 mg/kg) but significantly highest at SMWPO (1617.2 mg/kg) (Tukey HSD, *p* < 0.0001). Pb was significantly lowest in KISGS (3.0 mg/kg) and significantly highest in KANSDS (9.0 mg/kg) (Tukey HSD, *p* < 0.0001). A significantly lowest mean Zn (33.1 mg/kg) was observed at KISGS, while a significantly highest Zn (96.8 mg/kg) was recorded at the SMWPO. Notably, sediment from the SMWPO streams receiving smelter waste effluents had significantly highest pooled mean concentrations of Cu and Zn (Tukey HSD, *p* < 0.0001).

Similarly, the seasonal distribution of metal concentrations in sediments significantly varied (Figure 3B). As was significantly lowest in the LWS (6.1 mg/kg) and highest in the DHS (7.9 mg/kg) (Tukey HSD, *p* < 0.0001). Cu concentration ranged from 413.8 mg/kg in the LWS to 558.6 mg/kg in DCS and DHS but was significantly lowest in the LWS (Tukey HSD, *p* < 0.0001). Pb was significantly higher in the DCS (7.2 mg/kg) (Tukey HSD, *p* < 0.0001) but decreased to 4.7 mg/kg in the LWS, while Zn significantly increased from 43.9 mg/kg in the DCS to 53.7 mg/kg in the DHS. As for Cu, Pb and Zn in *Typha domingensis* .

#### 3.2.2 As, Cu, Pb and Zn in *Typha domingensis*

In Figure 2b and Table A5 in Appendix 1, As concentration in *Typha* ranged between 2.5 mg/kg at KYAFDS in the DHS and 7.7 mg/kg at KABR in the LWS. For Cu, the lowest concentration (5 mg/kg) was at KISDS, while the highest concentration (332 mg/kg) was in *Typha* from the SMWPO stream during the DHS. Pb concentration in *Typha* was lowest in KISDS (15.1 mg/kg) in the DCS and highest at KYABR (47.1 mg/kg) in the LWS. Zn ranged from 23.9 mg/kg at KYAFDS during the DCS to 104.8 mg/kg at KYABR in the LWS.

**FIGURE 4:**
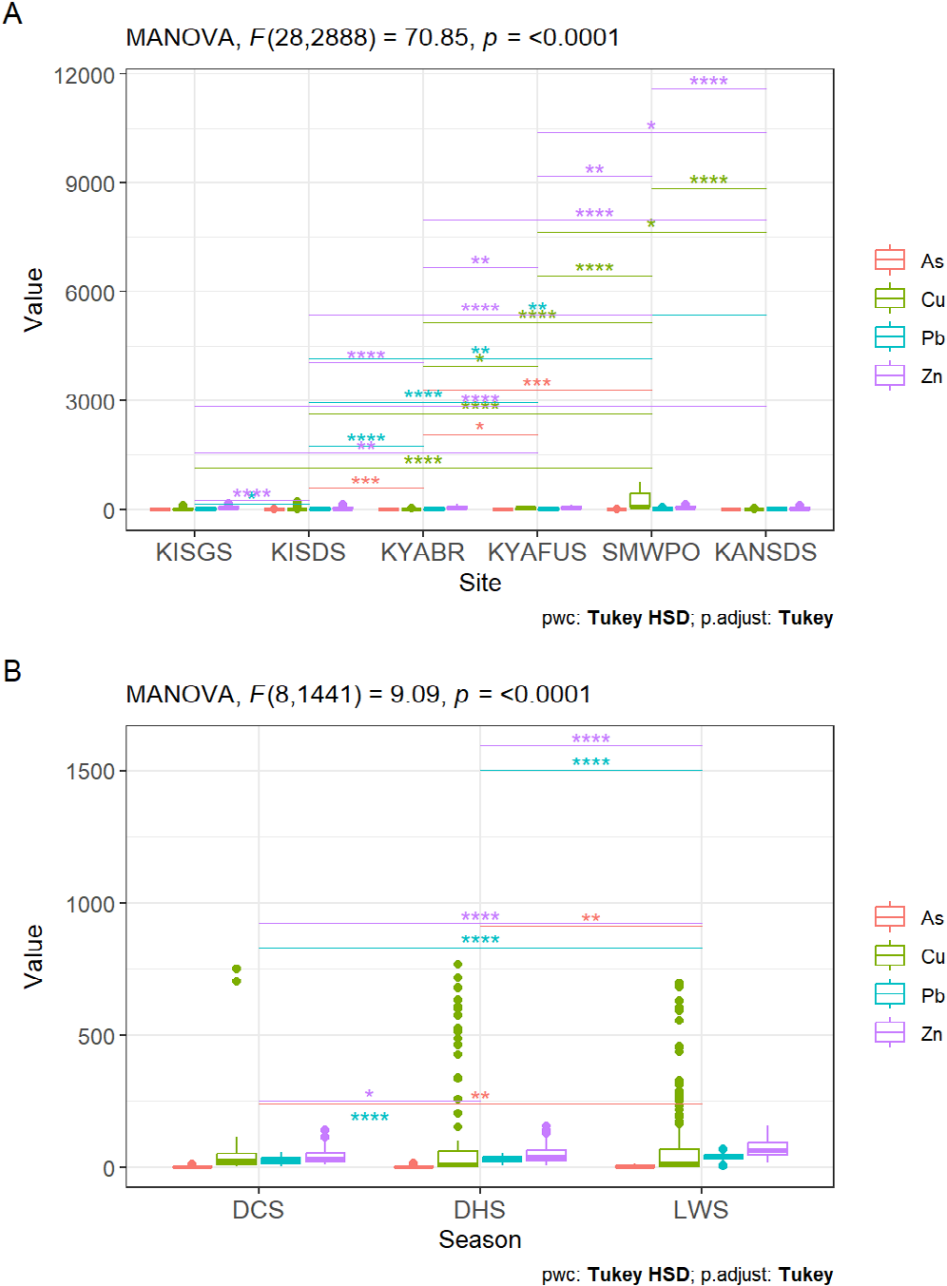
Tukey HSD post hoc tests for site (4A)‐ and season (4B)‐specific variances in metal concentrations in *Typha domingensis* biomass in the KSC streams. *Note:* *, **, *** and **** represent significant differences at *p* < 0.05, 0.01, 0.001 and 0.0001 respectively.

In Figure 4A, the pooled spatial variability of As concentration in *Typha* ranged from 3.0 mg/kg at KYAFDS to a significantly higher 5.5 mg/kg As at KYABR (Tukey HSD, *p* < 0.0001). Cu ranged from 8.8 mg/kg at KANSDS to a significantly highest concentration (258.6 mg/kg) at SMWPO (Tukey HSD, *p* < 0.0001); while Pb varied from 24.9 mg/kg at KYAFDS to a significantly higher concentration (36.0 mg/kg) at KYABR (Tukey HSD, *p* < 0.0001). The Zn in *Typha* ranged between 38.1 mg/kg at KYAFDS and a significantly highest concentration (74.7 mg/kg) at SMWPO. *Typha* biomass sampled from the SMWPO stream also had the highest Cu and Zn concentrations, while Pb was highest at KYABR (Tukey HSD, *p* < 0.0001).

The overall seasonal distribution of As, Cu, Pb and Zn in *Typha* biomass in the KSC varied significantly (Figure 4B). As was lowest in the DCS (3.7 mg/kg), slightly increased in the DHS (3.84 mg/kg) and significantly peaked at 4.7 mg/kg in the LWS (Tukey HSD, *p* < 0.0001). Cu was lowest in the DCS (39.7 mg/kg) but significantly higher in the DHS (62.6 mg/kg). Pb and Zn concentrations were also lowest in the DCS (25.4 and 40.4 mg/kg, respectively) but significantly increased in the LWS (37.8 and 70.7 mg/kg, respectively) (Tukey HSD, *p* < 0.0001).

### 3.3 Stream Sediment Quality and Metal Toxicity Profiles

**FIGURE 5:**
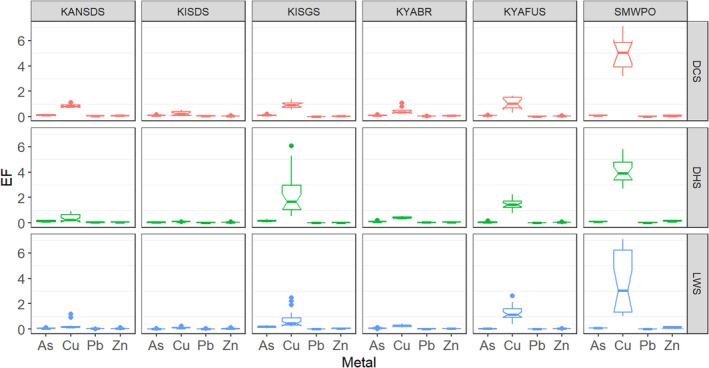
Spatial and seasonal variability in enrichment with As, Cu, Pb and Zn of surface sediments of KSC streams.

Figure 5 and Table A4 in Appendix 1 detail the EF values to establish the magnitude of sediment enrichment with As, Cu, Pb and Zn in KSC streams. Considering the sites, the sediments were moderately enriched with Cu at SMWPO (EF 3.92–5.01) while KYAFUS (EF 1.02–1.45) and KISGS (EF 0.75–2.19) recorded minor Cu enrichment, indicating a considerable degree of anthropogenic contribution at SMWPO and KISGS streams. EF values of 0.5–1.5 indicate natural sources, while EF > 1.5 imply anthropogenic contribution. However, no notable sediment enrichment was observed for AS, Pb and Zn (EF < 1) at all six streams. The overall seasonal sediment enrichment in KSC followed the order Cu (1.47) > As (0.10) > Zn (0.05) > Pb (0.04) in the DCS; Cu (1.37) > As (0.10) > Zn (0.06) and Pb (0.05) for the DHS; and Cu (1.03) > As (0.08) > Zn (0.06) > Pb (0.01) in the LWS.

**FIGURE 6:**
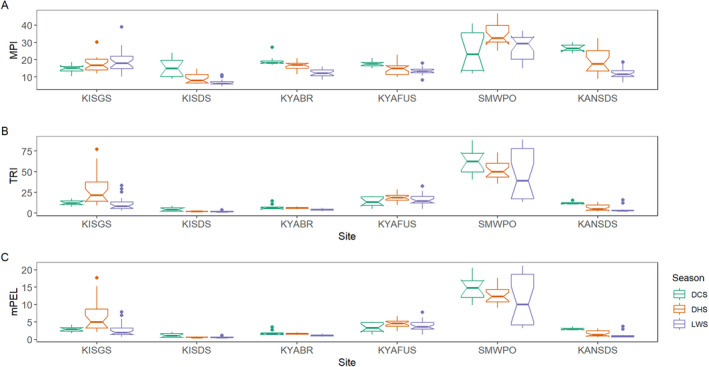
Spatiotemporal trends in stream sediment quality assessment indices: (A) MPI (metal pollution index), (B) TRI (toxic risk index) and (C) mPEL (mean probable effect level) for the KSC streams.

In Figure 6 and Table A4 in Appendix 1, the MPI, TRI and mPEL values were consistently higher at the SMWPO sediments across the seasons in agreement with the EF results (Figure 5). In the DCS, KANSDS (26.85) and SMWPO (24.99) recorded the two highest MPI (Figure 6A). However, MPI was highest in SMWPO sediments in the DHS (34.74) but slightly decreased in the LWS (27.34). The site MPI ranked in the order KANSDS > SMWPO > KYABR > KYAFUS > KISDS > KISGS in the DCS, SMWPO > KANSDS > KISGS > KYABR > KYAFUS > KISDS, and SMWPO > KISGS > KYAFUS > KYABR > KANSDS > KISDS during the LWS. Seasonally, the MPI followed the order DCS (19.23) > DHS (18.09) > LWS (14.90). Considering the MPI = 1 threshold value, therefore, the stream sediments in all the sites were contaminated with As, Cu, Pb and Zn, with Cu being the significant contributor as observed in the EF trends (Figure 5 and Table A4 in Appendix 1).

Similar to MPI, the TRI indicated highest values at the SMWPO (TRI 62.52–52.16–49.62) generally indicating a “very high” toxic risk (TRI > 20) at the site over the three seasons (Figure 6B). KISDS had the lowest TRI (4.54–2.20–1.99) which implied a “low” toxic risk classification (TRI ≤ 5) across the three seasons (Table A2 in Appendix 1). In addition, a gradual decrease in TRI from DCS to LWS was observed at both sites. The overall seasonal TRI in the sediments decreased from 19.35 to 18.26 to 13.78 in the DCS, DHS and LWS respectively, indicating ‘moderate‐to‐considerable’ toxic risk in the sediments (10 < TRI ≤ 20).

In Figure 6C, the mPEL was likewise slightly higher at SMWPO (14.86) in the DCS but relatively lower but stable in the DHS (12.78) and LWS (12.03). Based on the mPEL classification (Table A2 in Appendix 1), the SMWPO sediments had a very high probability (> 73%) of toxicity to aquatic biota (mPEL > 2.5). In contrast, the mPEL was lowest at KISDS, ranging from 4.54 to 1.99 over the three seasons, indicating moderate‐to‐high probability (49%–73%) of toxicity (mPEL > 1.5–2.3). The KSC pooled seasonal mPEL decreased in the order DCS (4.65) > DHS (4.44) > LWS (3.39) with a 73% probability of toxicity (mPEL > 2.3) for the stream sediments in the catchment.

### 3.4 Bioaccumulation and Translocation of Metals by *Typha domingensis*

#### 3.4.1 Bioaccumulation Efficiency

**FIGURE 7:**
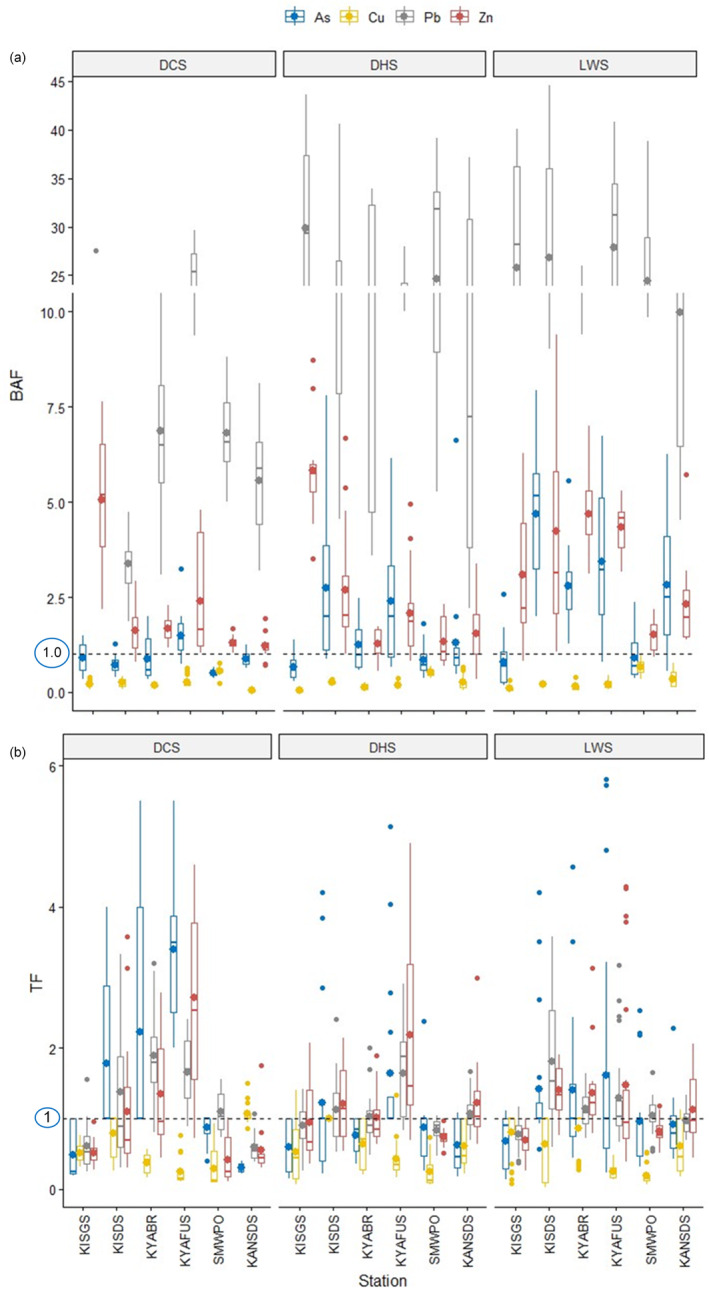
(a) Bioaccumulation factor (BAF) and (b) translocation factor (TF) of *Typha domingensis* for As, Cu, Pb and Zn contamination in streambed sediments in KSC, northwestern Zambian Copperbelt.

In Figure 7a and Table A6 in Appendix 1, the mean BAF in *T. domingensis* for As ranged from 0.51 at SMWPO to 4.68 at KISDS during the LWS. All the streams had peak BAF > 1 except for KISGS for all seasons, KISDS, KYABR and SMWPO in the DCS. In contrast, BAF < 1 was reported in *Typha* for Cu, from 0.06 at KANSDS in the DCS to 0.67 at SMWPO during the LWS. However, *Typha* indicated a higher bioaccumulation potential for Pb with BAF > 1 values from 0.35 at KANSDS in the LWS to 29.8 at KISGS in the DHS. The BAF for Zn was lowest (1.23) at KANSDS in the DCS and highest (5.8) at KISGS in the DHS, indicating a moderate bioaccumulation potential by *Typha* for Zn.

Considering the overall spatiotemporal variability of bioaccumulation efficiency for metals (Table A6 in Appendix 1), As in *Typha* at all streams except KISGS and SMWPO had BAF > 1. The BAF ranged from 1.62 to 2.53, implying the potential of *Typha* to remove As from the sediments in a majority of the streams. Seasonally, the BAF for As in the DCS was low (0.93) but above 1 for DHS and LWS (1.59–2.37). The BAF was very low for Cu (BAF < 1) in all the sites and seasonally. Nevertheless, the bioaccumulation potential for Pb was up to 24‐fold higher in all the streams (BAF 10.7–24.3) and up to 22‐fold seasonally (BAF 10.5–22.9), depicting hyperaccumulation potential for the metal. *Typha* also indicated a moderate bioaccumulation potential for Zn spatially (BAF 1.68–4.32) and seasonally (BAF 2.21–3.38).

#### 3.4.2 Translocation Efficiency

In Figure 7b, the TF in *Typha* for As ranged from 0.3 at KANSDS to 2.22 at KYABR in the DCS. TF for Cu was from 0.19 at SMWPO in the LWS to 1.07 at KANSDS in the DCS. *Typha* translocated Pb in the magnitude of 0.59 at KANSDS to 1.90 at KYABR during the DCS and Zn from TF 0.41 at SMWPO to TF 2.71 at KYAFUS in the DCS.

Generally, *Typha* stabilised As at KISGS, SMWPO, and KANSDS (TF 0.59–0.91) but extracted the metalloid at KISDS, KYABR, and KYAFUS (TF 1.47–2.08) in the DCS and LWS (Table A7 in Appendix 1). *Typha* mostly extracted As in the DCS and LWS (TF > 1) but stabilised the metalloid in the DHS (TF 0.95). *T. domingensis* phytostabilised Cu in all the stations and throughout the sampling seasons (TF < 1). Pb was more rapidly transferred at KISDS, KYABR, and KYAFUS (TF > 1) but rather stabilised at KISGS, SMWPO, and KANSDS (TF < 1). However, the seasonal Pb trend indicated the phytoextraction ability of *Typha* (TF 1.09–1.22). Similarly, Zn was extracted in all the streams (TF 1.24–2.0) except KISGS and SMWPO, where phytostabilisation was evident (TF < 1). Additionally, Zn transfer by *Typha* was at the TF > 1 magnitude in all three seasons.

#### 3.4.3 Metal Accumulation Index (MAI)

Table 2 gives the bioaccumulation potential as determined by MAI for *T. domingensis* for As, Cu, Pb and Zn from the stream sediments and in comparison with other macrophyte species in aquatic ecosystems elsewhere. The MAI ranged between 1.5 in KISDS and 3.9 at KYABR, all occurring during the LWS. The pooled site MAI ranked as follows: SMWPO > KYABR > KANSDS > KYAFUS = KYAFDS > KISGS > KISDS, with MAI > 2.0 in 57% of the streams. The trend is consistent with the MPI, TRI and mPEL results obtained in Section 3.3, further indicating the influence of high Cu content in the stream sediments on metal bioaccumulation in *T. domingensis* .

**TABLE 2:** Spatiotemporal metal accumulation index (MAI) for *Typha domingensis* in the KSC streams and comparison with selected macrophyte species in different aquatic systems.

Macrophyte	Location	Season	Metal accumulation index (MAI)							References
			KISGS	KISDS	KYABR	KYAFUS	KYAFDS	SMWPO	KANSDS	
*T. domingensis*	KSC, Zambia	DCS	1.9	1.8	2.0	2.0	1.9	2.2	2.2	Current study
		DHS	1.9	1.6	1.9	1.7	2.3	2.8	2.1	
		LWS	2.0	1.5	3.9	2.6	2.0	3.0	2.8	
		**Mean**	**1.9**	**1.6**	**2.6**	**2.1**	**2.1**	**2.7**	**2.3**	
*E. crassipes*	Lake Idku, Egypt	Mean	23.7							El‐amier et al. (2018)
*E. stagnina*		Mean	14.4							
*P. australis*		Mean	56.2							
*T. domingensis*		Mean	19.1							
*L. minor*	Rzuchowska River, Poland	Mean	1.50							Polechońska et al. (2022)
*L. trisulca*		Mean	2.33							
*T. latifolia*	Bahmansir River, Iran	Mean	3.08							Sasmaz et al. (2008), Haghnazar et al. (2021)
		Mean	4.82							
*T. latifolia*	5 ponds, Olesno‐Poland	Mean	2.76							Klink et al. (2013)
*T. latifolia*	River estuary, Catalonia‐Italy	Mean	8.21							Bonanno et al. (2018)

*Note:* The values in bold indicate the respective overall seasonal mean metal accumulation index (MAI) for *T. domingensis* sampled from the different streams.

Abbreviations: Macrophyte species: *E. crassipes* , *Eichhornia crassipes* ; *E. stagnina* , *Echinochloa stagnina* ; *L. minor* , *Lemna minor* ; *L. trisulca* , *Lemna trisulca* ; *P. australis* , *Phragmites australis* ; *T. domingensis* , *Typha domingensis* ; *T. latifolia* , *Typha latifolia* . Seasons: DCS, dry cold season; DHS, dry hot season; LWS, long wet season. Streams: KANSDS, Kansanshi downstream; KISDS, Kisengisengi downstream; KISGS, Kisengisengi source; KSC, Kansanshi sub‐catchment; KYAFDS, Kyafukuma downstream; KYAFUS, Kyafukuma upstream; SMWPO, Smelter waste pond outlet.

The pooled MAI for the streams is comparable with values reported for two floating‐leaved duckweed taxa, *Lemna minor* and *L. trisulca* in Poland's Rzuchowska River (MAI 1.5–2.33) and the emergent *Typha latifolia* in Olesno region wetland ponds (MAI 2.76). However, the MAI values for *T. latifolia* in Bahmansir River, Iran (MAI 4.82) and Catalonia River estuary, Italy (MAI 8.21) were 17% to 30% higher than those obtained in the KSC. Much higher MAI values were obtained in Lake Idru, Egypt for the floating‐leaved water hyacinth, *Eichhornia crassipes* (MAI 23.7) and two perennial wetland grasses, *Echinochloa stagnina* (MAI 14.4) and *Phragmites australis* (MAI 56.2). The MAI for *T. domingensis* in the same Lake was almost 70% higher than the value reported in this study. However, it is important to note that the MAI values were determined under different environmental conditions and different metal(loid)s were considered in each scenario (Bonanno et al. 2018; El‐amier et al. 2018; Haghnazar et al. 2021; Klink et al. 2013; Polechońska and Klink 2023; Sasmaz et al. 2008).

### 3.5 Multivariate Analysis

From the MANOVA test results (Table 3 and Table A8 in Appendix 1), both site (Pillai's Trace = 2.32, *F*_28,1348_ = 66.18, *p* < 0.05, $\eta_{p}^{2}$ = 0.58) and season (Pillai's Trace = 0.26, *F*_8,670_ = 12.30, *p* < 0.05, $\eta_{p}^{2}$ = 0.13) exhibited substantial impacts on the metal concentrations in the sediments. Additionally, the interaction effect between site and season was significant (Pillai's Trace = 0.62, *F*_44,1348_ = 5.56, *p* < 0.05, $\eta_{p}^{2}$ = 0.15), suggesting the joint influence of site and season on the variability of metal concentrations in the sediments. Similar significant differences were observed for site (Pillai's Trace = 1.63, *F*_28,1348_ = 70.85, *p* < 0.05, $\eta_{p}^{2}$ = 0.41) and season (Pillai's Trace = 0.32, *F*_44,1348_ = 34.27, *p* < 0.05, $\eta_{p}^{2}$ = 0.16) and the joint influence of site and season (Pillai's Trace = 0.53, *F*_44,1348_ = 9.09, *p* < 0.05, $\eta_{p}^{2}$ = 0.13) on metal concentrations in *T. domingensis* .

**TABLE 3:** Two‐factor multivariate analysis of variance (MANOVA) for spatiotemporal variability of As, Cu, Pb, and Zn in sediment and *Typha domingensis* , BAF, and TF for As, Cu, Pb and Zn by *T. domingensis* from streams in the Kansanshi sub‐catchment.

Factor	Pillai's statistic	*F*	num DF	den DF	*p*	$\eta_{p}^{2}$
Sediment						
Site	2.32	66.18	28	1348	< 0.0001***	0.58
Season	0.25	12.30	8	670	< 0.0001***	0.13
Site × Season	0.62	5.56	44	1348	< 0.0001***	0.15
*Typha domingensis*						
Site	1.63	70.85	28	2888	< 0.0001***	0.41
Season	0.32	34.27	8	1441	< 0.0001***	0.16
Site × Season	0.53	9.09	48	2888	< 0.0001***	0.13
Bioaccumulation factor (BAF)						
Site	2.13	51.84	24	1088	< 0.0001***	0.53
Season	0.45	19.77	8	540	< 0.0001***	0.23
Site × Season	0.92	8.14	40	1088	< 0.0001***	0.23
Translocation factor (TF)						
Site	1.49	29.94	24	1208	< 0.0001***	0.37
Season	0.12	4.77	8	600	< 0.0001***	0.06
Site × Season	0.54	4.71	40	1208	< 0.0001***	0.13

*** represents significance at *p* < 0.001.

At least one of the BAF for metals in *Typha* varied significantly among the sites (Pillai's Trace = 2.13, *F*_24,1088_ = 51.84, *p* < 0.05, $\eta_{p}^{2}$ = 0.53) and seasons (Pillai's Trace = 0.45, *F*_8,540_ = 19.77, *p* < 0.05, $\eta_{p}^{2}$ = 0.23) while the joint effect of site and season (Pillai's Trace = 0.92, *F*_40,1088_ = 8.14, *p* < 0.05, $\eta_{p}^{2}$ = 0.23) also markedly influenced the differences in BAF of *T. domingensis* . Similarly, the differences in TF for metals in *T. domingensis* were spatially (Pillai's Trace = 1.49, *F*_24,1208_ = 29.94, *p* < 0.05, $\eta_{p}^{2}$ = 0.37) and temporally (Pillai's Trace = 0.12, *F*_8,600_ = 4.77, *p* < 0.05, $\eta_{p}^{2}$ = 0.06) significant. Moreover, the interaction effect of site and season for variability in metal translocation was also substantial (Pillai's Trace = 0.54, *F*_40,1208_ = 4.71, *p* < 0.05, $\eta_{p}^{2}$ = 0.13), emphasising the joint spatial and temporal influence of metal transfer by *T. domingensis* .

As factors with $\eta_{p}^{2}$ values ≥ 0.14 (14%) are considered to have a larger effect size (Richardson 2011), site differences accounted for a higher variance in the differences in metal concentrations in sediments and *T. domingensis* (37%–58%). Differences due to seasons accounted for 6%–23%, whereas the joint effect of site and season explained 13%–15% of the variance in metal(loid) levels in the sediment and *T. domingensis* , and BAF and TF values for the macrophyte (Table A7 in Appendix 1).

### 3.6 Linear Discriminant Analysis

#### 3.6.1 Stream Sediments

**FIGURE 8:**
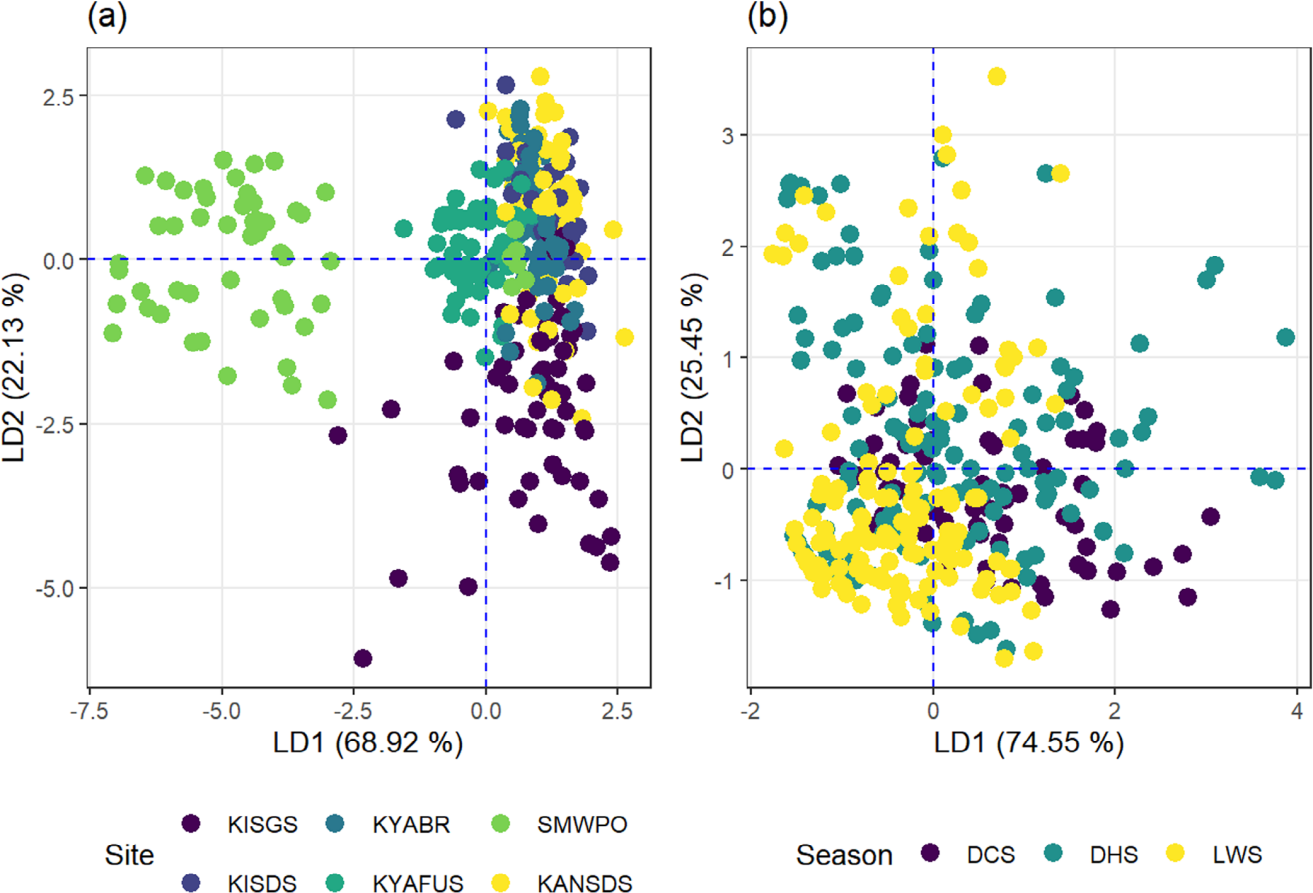
Linear discriminant analysis biplots for (a) spatial and (b) seasonal distribution of metals in the bed sediments of the KSC streams.

A MANOVA post hoc test, the linear discriminant analysis (LDA) was conducted to isolate the specific site and seasonal differences in the joint effect of metal concentrations on stream sediment quality. The LDA generated four discriminant functions (LD) for site comparisons. LD1 and LD2 accounted for up to 89.05% of the variability in metal concentrations in the sediments (Figure 8a). The LD1 discriminant function was defined as *Y*_LD1Site_ = 0.104As − 0.003Cu + 0.013Pb − 0.024Zn. The contamination of sediments in the different sites followed the order As > Zn > Pb > Cu. Furthermore, As and Pb jointly contributed positively, although Cu and Zn jointly negatively impacted sediments in at least one of the streams. In LD1, As (4.2 mg/kg) and Pb (2.96 mg/kg) were lowest at KYAFUS and KISGS, respectively. However, Cu (1617 mg/kg) and Zn (96.79 mg/kg) were highest at the SMWPO. Therefore, LD1, explaining 68.92% of the variability in contamination, isolated the SMWPO stream sediments as most contaminated with Cu and Zn. Similarly, the KYAFUS and KISGS were also relatively well separated, although with some overlaps, and exhibited less contamination of sediments, particularly with As and Pb.

Seasonally, the LD1 and LD2 functions explained 74.55% and 25.45%, respectively, the differences in the overall stream sediment quality in relation to metal concentrations (Figure 8b). Considering seasonal patterns, the LD1 function: *Y*_LD1Season_ = 0.049As + 0.001Cu + 0.274Pb − 0.015Zn, As, Cu and Pb jointly contributed positively to stream sediment quality, whereas the negative contribution was mainly attributed to Zn. Sediment contamination by metal(loid) followed the order Pb > As > Zn > Cu. The LWS exhibited the lowest concentrations of As (6.16 mg/kg), Cu (389.3 mg/kg), and Pb (4.44 mg/kg), indicating reduced contamination by these elements during the LWS.

#### 3.6.2 *Typha domingensis*

**FIGURE 9:**
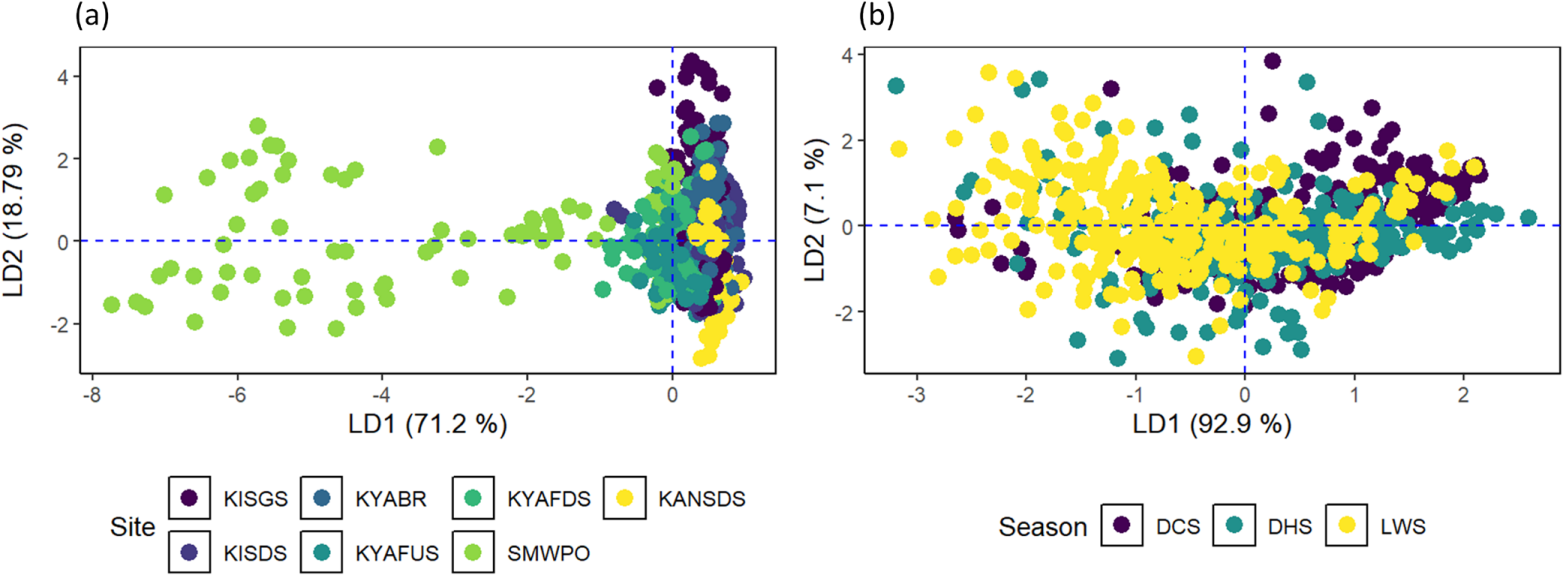
Linear discriminant analysis biplots for (a) site and (b) seasonal variability of the joint effect of As, Cu, Pb and Zn concentrations in *Typha domingensis* in the KSC streams.

The LDA for site differences generated LD1 (71.2%) and LD2 (18.8%) to explain the distribution of meta(loid) concentrations in *T. domingensis* (Figure 9a). From the spatial discriminant function LD1_Site_: 0.044As − 0.012Cu − 0.02Pb + 0.002Zn, As and Zn jointly contributed positively, but Cu and Pb jointly negatively influenced metal concentrations in *Typha*. Generally, the metal concentrations in the plant tissue decreased in the order As > Pb > Cu > Zn. Considering LD1, As (3.71 mg/kg) and Zn (39.6 mg/kg) were lowest in *T. domingensis* at the SMWPO and KANSDS streams, respectively. In contrast, Cu (258.6 mg/kg) and Pb (69.4 mg/kg) were highest overall in the *T. domingensis* biomass sampled at the SMWPO stream, which implies higher sediment contamination with Cu and Zn, as similarly observed in Section 3.4.1 in this study.

In Figure 9b, the LD1 accounted significantly (92.9%) for seasonal differences in metal(loid) concentrations in *Typha* biomass. The seasonal LD1 function LD1_season_: 0.204As + 0.0004Cu − 0.056Pb − 0.026Zn defined the joint positive contribution of As and Cu, while Pb and Zn jointly contributed negatively to metal concentrations in *T. domingensis* . The lowest concentrations of As (3.7 mg/kg) and Cu (39.7 mg/kg) in *T. domingensis* were observed during the DCS, even though high Pb (37.8 mg/kg) and Zn (70.4 mg/kg) concentrations were in the LWS. In Figure 9b, the contrasting seasons were relatively well separated, with LWS biased towards the negative values on the horizontal plane.

### 3.7 Bioaccumulation and Translocation Efficiency for Metal(loid)s by *T. domingensis*

Considering bioaccumulation and translocation as phytoremediation strategies for *T. domingensis* , positive contributions of individual metals to the LDA functions imply high efficiency and vice versa.

#### 3.7.1 Bioaccumulation Factor (BAF)

**FIGURE 10:**
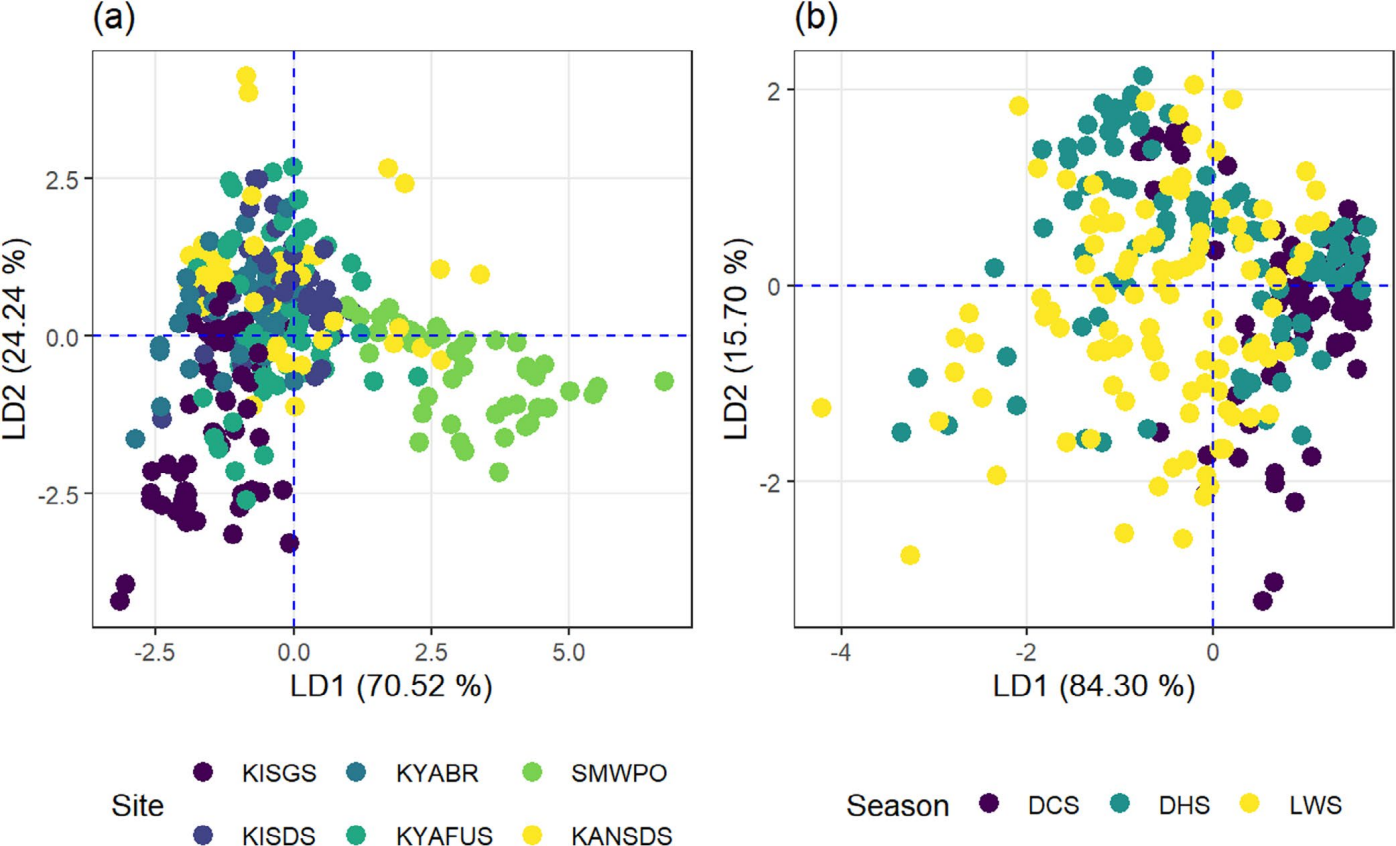
(a) Spatial and (b) temporal discrimination of the joint bioaccumulation factor (BAF) for As, Cu, Pb, and Zn by *Typha domingensis* in the KSC streams.

The discrimination of joint BAF for metal(loids) by *Typha* for the different sites as defined by the LDA is shown in Figure 10a, where LD1 accounts for 70.52% and LD2 explains 24.24% of the spatial variability in BAF. The linear function for site separation, LD1_Site_: −0.005As + 7.75Cu + 0.04Pb − 0.375Zn consisted of As and Zn contributing negatively, whereas Cu and Pb contributed positively to the joint BAF for metals by *Typha*. The bioaccumulation followed the order Cu > Zn > Pb > As. *Typha* was most efficient at accumulating Cu (0.58) and Pb (24.33) at the SMWPO and KISGS streams, respectively. In contrast, *Typha* had low bioaccumulation for As (0.79) and Zn (1.4) at the SMWPO stream.

Figure 10b illustrates the seasonal discrimination of BAF for metal(loid)s by *Typha*, where LD1 and LD2 represent 84.3% and 15.7% of seasonal differences, respectively. The function LD1_Season_: −0.376As − 0.967Cu − 0.078Pb + 0.045Zn describes negative joint contributions by As (2.37), Cu (0.27) and Pb (22.92) in the LWS, while Zn (2.21) jointly contributed positively in the DCS to BAF efficiency by *Typha*. The joint BAF order of contribution by metals seasonally to LD1 followed the order Cu > As > Pb > Zn, deviating from the metal BAF order for *T. domingensis* biomass at the spatial scale.

#### 3.7.2 Translocation Factor (TF)

**FIGURE 11:**
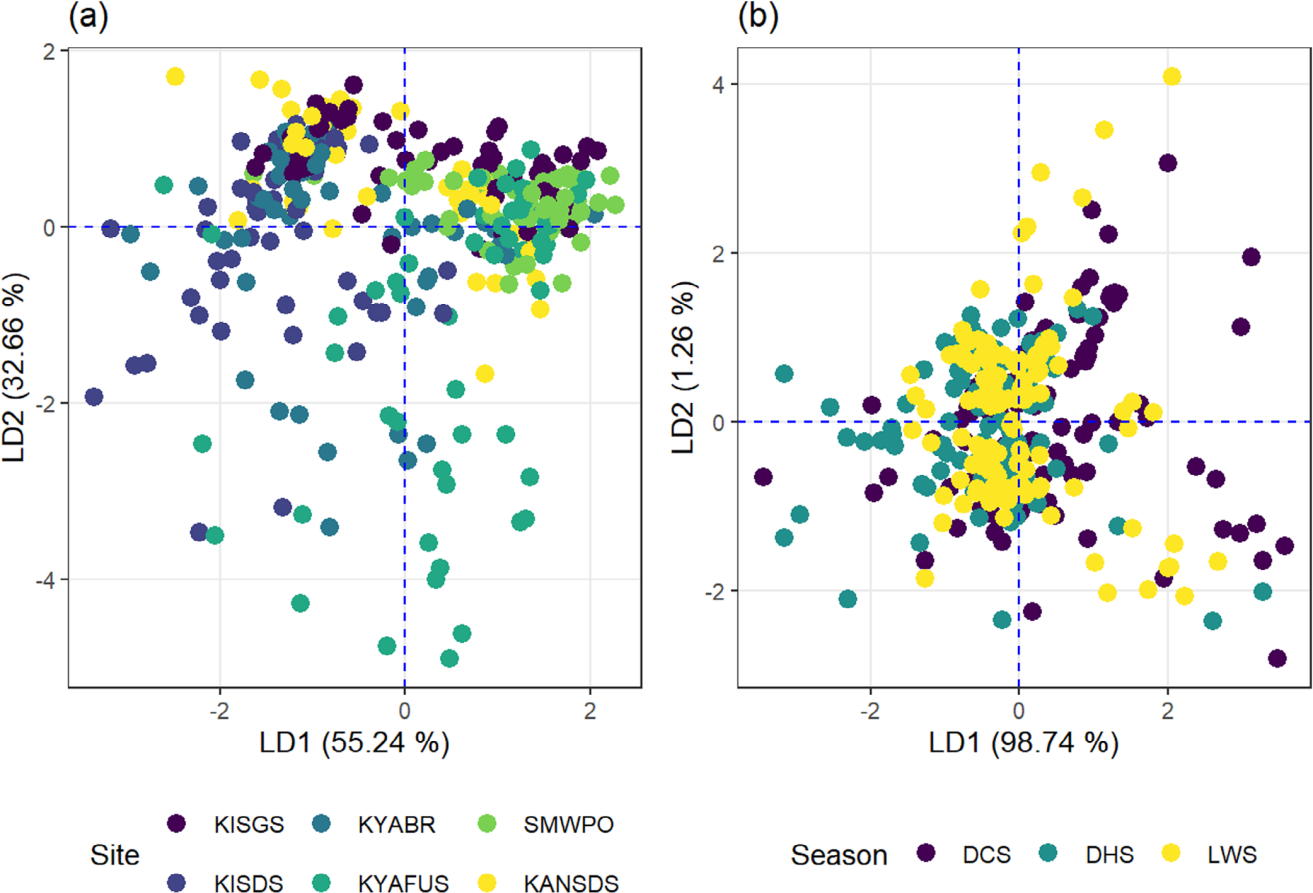
Linear discriminant analysis biplots for (a) spatial and (b) temporal variability of the translocation efficiency (TF) for As, Cu, Pb and Zn by *Typha domingensis* in the KSC streams.

The LDA for TF generated four Pillai's trace proportions where LD1 and LD2 achieved 55.2% and 32.7% separation for sites, respectively (Figure 11a). In the discriminant function LD1_site_: −0.133As − 3.474Cu − 1.521Pb + 0.313Zn, except for Zn, the remaining elements jointly contributed negatively to the metal translocation efficiency by *Typha*. The trend of elemental contribution to the LD1 function was Cu > Pb > Zn > As. The TF values for As (0.59) and Pb (0.76) were lowest at KISGS, while the translocation of Cu (0.23) was lowest at SMWPO. However, *T. domingensis* most efficiently transferred Zn (1.49) to the aerial parts at the KYAFUS stream.

Seasonally, the function LD1 separated a significant proportion (98.7%) of the joint TF for the elements by *Typha* (Figure 11b). From LD1_season_: 1.03As + 0.12Cu + 0.83Pb − 1.46Zn, As, Cu and Pb jointly contributed positively, but Zn had a negative influence on the seasonal variability in the translocation efficiency by *T. domingensis* . The TF was highest for As (1.51) and Pb (1.22) in the DCS. For Cu, the translocation efficiency (TF 0.57) peaked in the DHS. The joint seasonal TF of metals by *Typha* followed the order Zn > As > Pb > Cu, which varied from the spatial trend where Cu showed the highest contribution to the overall TF.

## 4 Discussion

### 4.1 Metal Distribution and Surface Sediment Quality in KSC Streams

In concordance with Kříbek et al. (2023) and Mata et al. (2020), the elevated Cu and Zn concentrations in sediments of the SMWPO stream were mostly associated with anthropogenic inputs, including mining and ore processing in the KSC. This has been demonstrated by the high EF for Cu, and high MPI, TRI and mPEL values of sediments at SMWPO in this study. Similar findings were also reported by Iordache et al. (2022) who revealed that the elevated concentration of Cu (up to 176 mg/kg) and Zn (up to 172.9 mg/kg) in Romania's Olt River sediments was attributed to anthropogenic activities, particularly mining. Also, in Zambia, high Cu in sediments of three streams, Mufulira, Uchi and Kabwe, draining tailings dams (Nabuyanda et al. 2022) and Kafue River (M'kandawire et al. 2017) suggested anthropogenic pollution. In the DRC's Congo River, agroindustrial, mining and municipal waste effluents elevated As, Cu, Zn and Pb concentrations in the sediments (Mata et al. 2020). However, Cu concentrations were also consistently high in the KSC streams, given that the catchment lies within the Cu‐rich CACB (Hitzman et al. 2012). The variability of As and Pb levels in the KSC streams was mostly associated with natural background leaching and pH‐redox conditions in sediments and overlying water (Cui et al. 2014; Huang et al. 2022). As, Cu, Pb, and Zn concentrations in stream sediments of KSC were higher during the dry seasons (DCS and DHS). In concordance with other studies, this variability suggests the influence of hydrology, terrestrial inputs and dilution effect, which modulate geochemical fluxes and metal concentrations in sediments (Huang et al. 2020; Maphanga et al. 2024; Vinothkannan et al. 2022).

Metals enter aquatic ecosystems from natural and anthropogenic sources, after which approximately 85% accumulate in surface sediments (Zhang et al. 2016). In the sediments, metals bind to the organic and inorganic components variously, including adsorption onto the surface of Fe–Mn oxy‐hydroxides, complexation with organic matter and integration into metal sulphides (Zhang et al. 2014, 2016). Furthermore, the occurrence and magnitude of different metals in sediments can also be influenced by the physicochemistry of the overlying water (Maphanga et al. 2024; Zou et al. 2024).

### 4.2 Metal Distribution in *Typha domingensis* Biomass and Phytoindication Potential

From this study, similar to other cattail species (Klink et al. 2013; Minkina, Fedorenko, Nevidomskaya, Pol'shina, et al. 2021; Sesin et al. 2021), *T. domingensis* proved to be an excellent phytoindicator of Cu, Pb and Zn pollution and moderately indicated As contamination in all the streams (Figure 7b; Table A6 in Appendix 1). Other studies have also demonstrated the phytoindication potential of *Typha* spp. for Cd, Cu, Pb, and Zn in Queensland, Australia (Cardwell et al. 2002) and Mn, Zn, Cd, Pb, Ni and Cu in Southwest Poland (Klink 2017). The KANSDS and SMWPO, receiving mine wastewater, had the highest Pb and Cu concentrations in *T. domingensis* biomass (Section 3.2.2). Our results concur with other studies reporting similar influences of anthropogenic contributions to elevated metal concentrations in streams (Hejna et al. 2020; Huang et al. 2020). Alternatively, the low As and Zn at the SMWPO and KANSDS, respectively, suggested natural background levels (Salomão et al. 2021). Besides spatial variability, seasonal differences also influenced metal distribution in *Typha*, with elevated Pb and Zn during the wet season, whereas low As and Cu occurred in the low‐flow season (Engloner et al. 2022; Fawzy et al. 2012; Munyai and Dalu 2023).

Metal concentrations in *Typha* biomass also correlated directly with fluctuations in concentrations in the sediments (Hejna et al. 2020; Maphanga et al. 2024). For instance, the seasonal mean concentrations of As, Cu, Pb and Zn in *Typha* were 1.4, 2.5, 2.7 and 0.3 times higher, respectively, compared to findings by Bonanno et al. (2017) due to variations in sediment contamination by metals. Dube et al. (2019) also confirmed the positive correlation between sediment metal concentrations and uptake by *T. domingensis* in Zimbabwe's Insukamini Reservoir ecosystem.

Generally, metal indicators accumulate and tolerate contaminants in their biomass to detectable concentrations without phytotoxic impairment (Polechońska and Klink 2023; Sheoran et al. 2011). However, like most bioindicators, certain intrinsic factors that may limit the bioindication potential for metal pollution by *T. domingensis* should be considered during biomonitoring evaluation (Klink et al. 2013). Such factors include the metabolic ability to weaken the biochemical effects of contaminants, disruption of expected responses caused by the interaction among the pollutants, and growth suppression resulting from metal pollution (Zhou et al. 2008). Additionally, excluder taxa release compounds to the sediments from the root zone, which are known to bind or chelate metal, inhibiting their mobility and toxicity (Munyai and Dalu 2023). Furthermore, the capacity of macrophytes to accumulate metals varies with ambient environmental conditions, seasonal cycles, regional and ecological classifications (Petrov et al. 2023).

### 4.3 Phytoremediation of As, Cu, Pb and Zn by *T. domingensis*

#### 4.3.1 Bioaccumulation Efficiency (BAF)

Except for Cu (BAF < 1), the bioaccumulation efficiency was high (BAF > 1) in 71% of the KSC streams and indicates the accumulator potential of *T. domingensis* for As, Pb and Zn (Eid, Galal, Sewelam, et al. 2020; Eid, Galal, Shaltout, et al. 2020). Contrary to findings by Dube et al. (2019), the BAF of *T. domingensis* for As, Cu, Pb and Zn in this study varied spatiotemporally. Moreover, the seasonal BAFs were generally high (> 1), especially in the DCS and LWS for As, Pb, and Zn, but exceptionally low (< 0.5) for Cu. The elevated bioaccumulation efficiency for As, Pb and Zn could result from the high enrichment of these elements in the sediments, with significant influence of hydroperiodicity as observed in Section 4.1 (Dubey et al. 2017; Huang et al. 2020; Vinothkannan et al. 2022).

Our findings concur with other studies that noted higher bioaccumulation of As, Cu, Pb and Zn from sediments by *Typha* spp. in response to higher metal concentrations in the sediments (Engloner et al. 2022; Fawzy et al. 2012; Vymazal and Březinová 2015). For instance, metal concentrations in sediments were higher in SMWPO and KANSDS streams during the DHS. Hence, Pb showed the highest BAF, up to 20 times above the threshold value spatially and temporally. Bonanno et al. (2017) also demonstrated similar responses for As, Cu, Pb and Zn in three *Typha* congenerics ( *T. latifolia* , *T. domingensis* , *T. angustifolia* ) with high concentrations and elevated BAF.

However, the low BAF for Cu, particularly in the KSC streams, suggests *T. domingensis* as an excluder for Cu (Basallote et al. 2023; Dube et al. 2019), which could be an inhibitory response against extreme sediment enrichment with Cu (1:6 biomass to sediment ratio) from mining waste effluent discharge (Mufarrege et al. 2021, 2014). This is further supported by the low overall MAI values reported in Section 3.4.3 of the current study compared to other works (Bonanno et al. 2018; El‐amier et al. 2018; Haghnazar et al. 2021; Klink et al. 2013; Polechońska and Klink 2023; Sasmaz et al. 2008). Additionally, the high concentration of metals in sediment does not always correspond to increased toxic effects and bioavailability (Besser et al. 2003; Hernández‐Crespo et al. 2012). Other studies have also observed different self‐defence mechanisms of macrophytes against metal toxicity, including hypertolerance (Khalid et al. 2017) and hyperexclusion (Alexandra et al. 2017).

#### 4.3.2 Translocation Efficiency (TF)

We observed *T. domingensis* 's potential to extract As, Pb and Zn from the sediments in half of the KSC streams (TF > 1), which were also less impacted by mining activities, confirming findings from related studies (Bonanno 2013; Hadad et al. 2018). For instance, Lominchar et al. (2015) observed high BAF (121–3168) and TF > 1 by *T. domingensis* for Hg removal from sediments in Valdeazogues River, Spain, which further asserts the bioindication and phytoextraction capability of *T. domingensis* for aquatic metal contaminants.

Conversely, *T. domingensis* stabilised As, Pb and Zn in the moderately‐ to heavily‐impacted streams, though Cu was stabilised in all the streams irrespective of their ecological status (TF < 1). Similar findings of metal stabilisation by *Typha* spp. in sediments were also reported by Klink et al. (2013). In a study by Hadad et al. (2018), Cr, Ni and Zn were effectively stabilised by *T. domingensis* following bioaccumulation from wetland sediments. However, seasonal changes defined *T. domingensis* predominantly as a phytoextractor for As, Pb and Zn (TF > 1) and only stabilised Cu (TF < 1). According to Hadad et al. (2018), the dual phytoremediation characteristics of *T. domingensis* observed in this study may be dependent on biotic factors, such as microbial and enzymatic; and environmental factors, such as temperature, pH and redox potential, among others. For instance, speciation, concentrations and interactions of metals in the root zone and microbial biochemical processes can alter their solubility and bioavailability in the sediments (Alford et al. 2010; Sood et al. 2012).

## 5 Conclusions

This study provides insights into the spatiotemporal response and phytopotential of *Typha domingensis* to indicate and remediate aquatic metal(loid) pollutants on the CACB mining landscape. *T. domingensis* spatiotemporally discriminated the sources and magnitude of metal pollution in the stream sediments. Furthermore, the high biouptake and translocation capacity for Pb and Zn, coupled with the restricted Cu transfer, depicted *T. domingensis* suitability for continuous phytoremediation of metal‐polluted lotic systems in the mineral‐rich CACB.

Metals in *T. domingensis* at most streams were mainly sequestered in the below‐ground biomass where the generally high BAFs for As, Pb and Zn indicated *T. domingensis* as an accumulator of these elements. Additionally, the high TFs for As, Pb and Zn demonstrated *Typha's* metal extraction efficacy. However, the study observed a low TF value for Cu, which inferred a limited translocation, and therefore characterising *T. domingensis* as a potential phytostabiliser for Cu in the CACB stream sediments. Similar seasonal BAF and TF characteristics by *T. domingensis* for the four metal(loid)s were also observed.

The dual role of *T. domingensis* as a potential phytoextractor and phytostabiliser positions the macrophyte as a versatile candidate for phytorestoration of metal‐impacted aquatic ecosystems. However, there is a need to design and implement timely harvesting and safe disposal of *T. domingensis* biomass to avert the macrophyte from being a source rather than a sequester of metal(loid)s. Moreover, the integration of several native macrophyte taxa with complementary phytorestoration potentials will augment the effectiveness of onsite nature‐based management of aquatic metal pollution on the CACB.

## Author Contributions

**Kennedy O. Ouma:** conceptualization (lead), data curation (lead), formal analysis (lead), funding acquisition (lead), investigation (lead), methodology (lead), project administration (supporting), visualization (lead), writing – original draft (lead), writing – review and editing (equal). **Agabu Shane:** conceptualization (supporting), data curation (supporting), formal analysis (supporting), investigation (supporting), methodology (supporting), supervision (equal), writing – original draft (supporting), writing – review and editing (equal). **Concillia Monde:** conceptualization (supporting), formal analysis (equal), funding acquisition (supporting), investigation (supporting), supervision (supporting), writing – original draft (supporting), writing – review and editing (supporting). **Stephen Syampungani:** conceptualization (equal), data curation (supporting), formal analysis (supporting), funding acquisition (supporting), investigation (supporting), methodology (supporting), project administration (lead), resources (supporting), supervision (lead), validation (equal), visualization (supporting), writing – original draft (equal), writing – review and editing (equal).

## Conflicts of Interest

The authors declare no conflicts of interest.

## Supporting information


Data S1.

Data S2.

Data S3.

Data S4.

## Data Availability

Key supporting data are provided as Appendix and Supporting Information (Data [Link], [Link]) available online. Any additional data that support the findings of this study are available from the corresponding author upon reasonable request.

## Appendix 1

**TABLE A1:** Geophysical and hydrological characteristics of the sampled streams in Kansanshi sub‐catchment (KSC) on the north‐western Zambian Copperbelt.

No.	Stream and Station ID	GPS location and elevation (m a.s.l)	Mean channel width and depth (m)	Mean velocity (m/s)	Bed‐sediment type	Riparian zone features	Adjacent land use
1	Kisengisengi source (KISGS)	12°7′14.3″ S	W: 0.5–1.0	0.20–0.52	Sandy	Emergent macrophyte; shrubs; open	Water abstraction; farming; groundwater source
		26°29′51.6″ E	D: 0.3–0.5				
		1335 m	Wadable				
2	Kisengisengi downstream (KISDS)	12°6′43.1″ S	W: 3–5	0.28–0.81	Sandy	Emergent macrophyte; trees; shrubs; 5% canopy	Water abstraction; farming
		26°29′53.6″ E	D: 0.5–1.0				
		1329 m	Wadable				
3	Kifubwa downstream (KIFDS)	12°10′32.5″ S	W: 10–12	0.51–1.80	Sandy	Grass; shrubs; open	Water abstraction; drains Kisengisengi and Kansanshi
		26°26′52.8″ E	D: 1.5–2.0				
		1310 m	Not wadable				
4	Kansanshi downstream (KANSDS)	12°8′50.9″ S	W: 2–3.5	0.21–0.64	Loamy‐sand	Emergent macrophytes; grass; open	Water abstraction; open to public
		26°27′41.4″ E	D: 0.5–0.8				
		1400 m	Wadable				
5	Kyansafwa stream (KYAST)	12°8′41.9″ S	W: 1–1.5	0.18–0.20	Loamy‐sand	Grass; shrubs; 30%–40% canopy	Restricted area; receives mine water outflow
		26°24′26.7″ E	D: 0.5				
		1366 m	Wadable				
6	Kyansafwa Bridge (KYABR)	12°8′55.0″ S	W: 1.5	0.26–0.30	Loamy‐sand	Emergent macrophytes; grass; open	Water abstraction
		26°24′24.6″ E	D: 0.5				
		1350 m	Wadable				
7	Kyafukuma upstream (KYAFUS)	12°4′33.7″ S	W: 3–5	0.50–0.59	Sandy	Emergent macrophytes; grasses; open	Restricted area; drains into wetland
		26°22′59.7″ E	D: 0.5				
		1358 m	Wadable				
8	Kyafukuma downstream (KYAFDS)	12°5′27.9″ S	W: 0.7–1.0	0.19–0.48	Bed‐rock	Emergent and floating macrophytes; 30%–40% canopy	Restricted wetland; outflow into Solwezi River
		26°23′4.4″ E	D: 0.5				
		1355 m	Wadable				
9	Smelter waste pond outlet (SMWPO)	12°5′8.2″ S	W: 1–1.3	0.26–0.69	Sandy‐loam	Emergent macrophytes; grass; trees; 50%–60% canopy	Restricted area; drains into wetland; outflow at KYAFDS
		26°23′30.8″ E	D: 0.6				
		1363 m	Wadable				

**TABLE A2:** Geochemical indices used to in the study for sediment quality and toxicity risks assessment of metal contamination in KSC streams, north‐western Zambian Copperbelt between May 2022 and April 2023.

Expression	Formula	Classification	References
Enrichment Factor (EF)	$\mathrm{EF}=\left(C_{i}/C_{\mathrm{Fe}}\right)/\left(B_{i}/B_{\mathrm{Fe}}\right)$ where: *C*_*i*_ = concentration of metal in sediment (mg/kg); *C*_Fe_ = concentration of Fe (mg/kg); *B*_*i*_ = local background concentration for metal (mg/kg), (As—33; Cu—1620; Pb—81; Zn—356) *B*_Fe_ = local background value for Fe (47,200 mg/kg)	EF ≤ 1 no enrichment; 1 < EF ≤ 3 minor enrichment; 3 < EF ≤ 5 moderate enrichment; 5 < EF ≤ 10 moderately severe enrichment; 10 < EF ≤ 25 severe enrichment; 25 < EF ≤ 50 very severe enrichment; EF > 50 extremely severe enrichment	Hoang et al. (2021), Key et al. (2001)
Metal Pollution Index (MPI)	MPI = (*M*_1_ × *M*_2_ × *M*_3_ × … × *M*_*n*_)^1/*n*^ where *M* = metal concentration (mg/kg); *n* = number of metals considered	MPI < 1 no contamination MPI = 1 background contamination MPI > 1 contamination	Elgendy et al. (2024)
Toxic Risk Index (TRI)	$\mathrm{TRI}=\sum_{i=1}^{n}{\mathrm{TRI}}_{i}={\left\{\left[{\left(C_{i}/{\mathrm{TEL}}_{i}\right)}^{2}+{\left(C_{i}/{\mathrm{PEL}}_{i}\right)}^{2}\right]/2\right\}}^{1/2}$ where: TRI_*i*_ = toxic risk index of a metal; *C*_*i*_ = concentration a metal; *n* = number metals; TEL_*i*_ = consensus‐based threshold effect level value, mg/kg, for a metal (As—9.79; Cu—31.6; Pb—35.8; Zn—121); PEL_i_ = consensus‐based probable effect level value, mg/kg, for a metal (As—33; Cu—149; Pb—128; Zn—459); and TRI = integrated toxic risk index of a sample	TRI ≤ 5 no toxic risk; 5 < TRI ≤ 10 low risk; 10 < TRI ≤ 15 moderate risk; 15 < TRI ≤ 20 considerable risk; TRI > 20 very high risk.	Zhang et al. (2016), MacDonald et al. (2000)
Mean Probable Effect Level (PEL_meanQ_)	${\mathrm{PEL}}_{\text{meanQ}}=\frac{\sum \left(C_{i}/{\mathrm{PEL}}_{i}\right)}{n}$ where: *C*_*i*_ = metal concentration in the sediment sample (mg/kg), PEL_*i*_ = Consensus‐Based probable effect level of toxicity for respective metal in sample (As—33; Cu—149; Pb—128; Zn—459); *n* = number of metal contaminants	PEL_meanQ_ ≤ 0.1 low probability (3%); 0.1 ≤ PEL_meanQ_ < 1.5 low‐to‐medium probability (21%); 1.51 ≤ PEL_meanQ_ ≤ 2.3 medium‐to‐high probability (49%); PEL_meanQ_ > 2.3 high probability (73%)	MacDonald et al. (2000), Long et al. (2006)
Metal Accumulation Index (MAI)	$\mathrm{MAI}=\frac{1}{n}\left(\sum_{j=1}^{n}I_{j}\right)$ where: *n* = total number of elements considered; *I*_*j*_ = *x*/*δx* is the sub‐index for variable *j* determined by dividing the mean value (*x*) by its standard deviation (*δx*)		Liu et al. (2007)

**TABLE A3:** Spatiotemporal distribution of metals in surface sediments of the Kansanshi sub‐catchment streams on the north‐western Zambian Copperbelt.

Season	Metal	KISGS	± SD	KISDS	± SD	KYABR	± SD	KYAFUS	± SD	SMWPO	± SD	KANSDS	± SD	Site pooled
DCS	As	9.3	2.3	7.6	2.3	7.6	1.7	5.8	1.3	7.1	1.4	9.8	2.3	7.3
DHS	As	13.0	4.9	4.9	2.9	7.6	3.1	4.3	2.9	8.2	1.8	10.4	4.7	7.9
LWS	As	14.7	5.6	2.3	0.7	5.1	1.9	3.3	1.5	7.0	1.5	4.7	1.9	6.1
Season pooled (As)		12.9		4.3		6.6		4.2		7.5		7.7		
DCS	Cu	342.5	96.7	96.2	69.7	172.7	89.6	386.1	177.0	1891.7	479.5	328.7	46.3	478.5
DHS	Cu	826.0	592.5	37.3	5.9	148.0	27.1	548.7	143.0	1594.5	408.2	148.8	106.6	558.6
LWS	Cu	281.6	241.7	41.7	20.4	97.8	30.3	478.7	200.9	1483.0	874.9	89.8	98.7	413.8
Season pooled (Cu)		511.5		50.8		132.9		487.2		1617.2		142.3		
DCS	Pb	2.5	0.0	10.2	3.2	8.4	1.4	5.3	3.1	4.7	2.5	12.5	2.9	7.2
DHS	Pb	2.5	0.0	5.0	2.8	6.4	3.3	2.5	0.0	4.9	3.0	9.6	5.3	6.2
LWS	Pb	3.7	1.6	3.4	1.7	5.2	2.2	3.3	1.3	3.4	1.3	7.5	2.6	4.7
Season pooled (Pb)		3.0		5.4		6.3		3.4		4.2		9.0		
DCS	Zn	23.5	8.5	38.3	14.0	55.7	12.3	46.0	12.3	50.4	50.3	53.8	7.1	45.8
DHS	Zn	22.8	16.6	37.9	10.2	55.8	21.7	38.1	15.1	126.1	38.3	49.9	16.8	53.7
LWS	Zn	48.3	18.1	35.2	18.4	42.4	10.5	30.7	5.8	93.9	56.1	37.6	19.9	45.9
Season pooled (Zn)		33.1		36.9		50.4		36.7		96.8		44.7		

Abbreviations: DCS, dry cold season; DHS, dry hot season; KANSDS, Kansanshi downstream; KISDS, Kisengisengi downstream; KISGS, Kisengisengi source; KYABR, Kyansafwa bridge; KYAFUS, Kyafukuma upstream; LWS, long wet season; SD, standard deviation; SMWPO, Smelter waste‐pond outlet.

**TABLE A4:** Spatiotemporal variability of selected sediment quality assessment factors and indices for metal in KSC streams during the May 2022–April 2023 period.

Season	Site	EF_As	± SD	EF_Cu	± SD	EF_Pb	± SD	EF_Zn	± SD	MPI	± SD	TRI	± SD	mPEL	± SD
DCS	KISGS	0.12	0.03	0.91	0.26	0.01	0.00	0.03	0.01	14.83	2.29	12.34	3.24	2.86	0.77
	KISDS	0.10	0.03	0.25	0.18	0.05	0.02	0.05	0.02	15.59	5.55	4.54	2.56	1.15	0.60
	KYABR	0.10	0.02	0.46	0.24	0.05	0.01	0.07	0.02	19.22	2.70	7.11	2.96	1.85	0.64
	KYAFUS	0.08	0.02	1.02	0.47	0.03	0.02	0.06	0.02	17.72	1.73	13.66	5.73	3.36	1.37
	SMWPO	0.09	0.02	5.01	1.27	0.03	0.01	0.06	0.06	24.99	12.27	62.52	15.08	14.80	3.32
	KANSDS	0.13	0.03	0.87	0.12	0.07	0.02	0.07	0.01	26.85	2.40	12.50	1.60	3.07	0.36
	**Overall**	**0.10**	**0.03**	**1.47**	**1.80**	**0.04**	**0.02**	**0.05**	**0.03**	**19.23**	**7.19**	**19.35**	**21.91**	**4.65**	**5.13**
DHS	KISGS	0.17	0.06	2.19	1.57	0.01	0.00	0.03	0.02	17.26	4.16	28.37	19.28	6.55	4.44
	KISDS	0.06	0.04	0.10	0.02	0.03	0.02	0.05	0.01	9.19	3.00	2.20	0.42	0.65	0.12
	KYABR	0.10	0.04	0.39	0.07	0.03	0.02	0.07	0.03	16.47	2.59	6.26	0.89	1.65	0.20
	KYAFUS	0.06	0.04	1.45	0.38	0.01	0.00	0.05	0.02	14.79	3.77	18.61	4.70	4.50	1.09
	SMWPO	0.10	0.02	4.10	0.95	0.02	0.01	0.15	0.05	34.74	6.32	52.16	11.53	12.78	2.62
	KANSDS	0.14	0.06	0.39	0.28	0.05	0.03	0.06	0.02	18.80	7.07	6.62	3.70	1.66	0.87
	**Overall**	**0.10**	**0.06**	**1.37**	**1.55**	**0.03**	**0.02**	**0.06**	**0.05**	**18.09**	**8.86**	**18.26**	**19.16**	**4.44**	**4.56**
LWS	KISGS	0.19	0.07	0.75	0.64	0.02	0.01	0.06	0.02	19.11	6.57	11.19	8.17	2.64	1.91
	KISDS	0.03	0.01	0.11	0.05	0.02	0.01	0.04	0.02	6.95	1.74	1.99	0.74	0.63	0.25
	KYABR	0.07	0.03	0.26	0.08	0.03	0.01	0.05	0.01	12.24	2.23	4.22	1.02	1.14	0.22
	KYAFUS	0.04	0.02	1.27	0.53	0.02	0.01	0.04	0.01	13.40	1.91	16.20	6.51	3.91	1.51
	SMWPO	0.09	0.02	3.92	2.32	0.02	0.01	0.11	0.07	27.34	7.14	49.62	28.50	12.03	6.76
	KANSDS	0.06	0.03	0.24	0.26	0.04	0.01	0.05	0.02	11.89	3.02	3.95	3.22	1.06	0.74
	**Overall**	**0.08**	**0.06**	**1.03**	**1.59**	**0.02**	**0.01**	**0.06**	**0.04**	**14.90**	**7.62**	**13.78**	**19.67**	**3.39**	**4.70**

*Note:* The values in bold indicate the respective overall mean (± SD) sediment quality index values across the three seasons.

Abbreviations for indices/factors: EF, enrichment factor; mPEL, mean potential effective level; TRI, Toxicity Risk Index.

**TABLE A5:** Spatiotemporal distribution of metal(loid)s in *Typha domingensis* from the KSC streams, north‐western Zambia.

Season	Metal	KISGS	± SD	KISDS	± SD	KYABR	± SD	KYAFDS	± SD	KYAFUS	± SD	SMWPO	± SD	KANSDS	± SD	Site pooled
DCS	As	4.3	3.3	2.8	1.8	3.2	2.8	2.7	1.6	4.6	3.1	4.7	3.3	4.5	2.7	3.7
DHS	As	4.4	3.2	4.3	3.0	5.7	4.4	2.5	1.3	2.9	2.2	3.2	1.9	4.4	2.9	3.8
LWS	As	4.5	3.4	3.9	2.9	7.7	2.9	3.7	3.1	5.0	2.8	3.4	2.2	5.0	2.6	4.7
Season pooled (As)		4.4		3.7		5.5		3.0		4.2		3.7		4.6		
DCS	Cu	47.2	28.2	6.4	3.5	13.9	8.6	37.4	15.1	48.9	33.9	129.8	176.4	8.7	4.1	39.7
DHS	Cu	20.0	18.4	5.0	0.0	8.5	6.6	45.2	35.2	48.5	26.1	332.1	251.5	8.4	5.3	62.6
LWS	Cu	8.6	11.3	28.2	47.6	6.1	3.4	74.3	46.7	44.4	29.9	295.5	215.9	9.3	6.9	62.1
Season pooled (Cu)		23.8		14.7		9.6		54.5		47.0		258.6		8.8		
DCS	Pb	21.6	8.9	15.1	9.1	28.0	11.0	15.4	9.0	37.9	11.7	30.4	6.2	33.3	11.2	25.4
DHS	Pb	32.2	11.6	32.5	10.3	31.5	11.1	24.5	7.6	25.1	10.1	36.6	8.5	32.5	10.1	30.4
LWS	Pb	40.3	9.8	33.0	16.4	47.1	7.0	32.3	9.8	41.9	9.4	35.0	6.6	34.9	6.8	37.8
Season pooled (Pb)		32.2		27.5		36.0		24.9		35.7		34.2		33.4		
DCS	Zn	57.1	27.4	28.2	13.6	41.0	15.3	23.9	12.6	53.3	30.2	45.1	26.5	33.1	15.3	40.4
DHS	Zn	75.4	33.9	50.0	26.2	49.2	24.0	27.4	9.3	31.5	24.8	70.5	18.4	33.0	17.7	46.9
LWS	Zn	67.3	40.1	59.9	29.6	104.8	24.6	56.8	28.7	67.2	24.6	87.6	22.2	55.9	21.1	70.4
Season pooled (Zn)		66.4		47.4		67.0		38.1		52.3		69.4		39.6		

Abbreviations: DCS, dry cold season; DHS, dry hot season; KANSDS, Kansanshi downstream; KISDS, Kisengisengi downstream; KISGS, Kisengisengi source; KYABR, Kyansafwa bridge; KYAFUS, Kyafukuma upstream; LWS, long wet season; SD, standard deviation; SMWPO, Smelter waste‐pond outlet.

**TABLE A6:** Bioaccumulation factors (BAF) by *Typha domingensis* for As, Cu, Pb and Zn in the Kansanshi sub‐catchment streams on the north‐western Zambian Copperbelt.

Season	Metal	KISGS	± SD	KISDS	± SD	KYABR	± SD	KYAFUS	± SD	SMWPO	± SD	KANSDS	± SD	Site pooled
DCS	As	0.92	0.41	0.73	0.23	0.86	0.56	1.50	0.59	0.51	0.10	0.87	0.18	0.93
DHS	As	0.67	0.34	2.74	2.19	1.25	0.73	2.41	1.77	0.85	0.41	1.30	1.40	1.59
LWS	As	0.79	0.62	4.68	1.89	2.79	1.01	3.44	1.80	0.92	0.54	2.83	1.78	2.37
Season pooled (As)		0.80		2.42		1.63		2.53		0.88		1.62		
DCS	Cu	0.22	0.10	0.25	0.11	0.18	0.04	0.27	0.14	0.57	0.14	0.06	0.03	0.25
DHS	Cu	0.05	0.03	0.26	0.03	0.14	0.08	0.19	0.07	0.52	0.10	0.28	0.19	0.26
LWS	Cu	0.10	0.06	0.20	0.03	0.15	0.10	0.21	0.10	0.67	0.17	0.35	0.22	0.27
Season pooled (Cu)		0.12		0.24		0.16		0.22		0.59		0.23		
DCS	Pb	17.23	3.77	3.37	0.84	6.86	2.11	21.69	7.15	6.81	1.21	5.56	1.58	10.48
DHS	Pb	29.82	9.51	20.13	11.59	18.71	14.31	20.11	5.11	24.65	12.71	14.68	13.79	21.00
LWS	Pb	25.80	10.77	26.85	12.75	16.98	5.24	27.91	9.16	24.39	7.73	0.35	0.22	22.93
Season pooled (Pb)		24.34		15.18		13.47		23.63		20.09		10.73		
DCS	Zn	5.05	1.82	1.62	0.54	1.67	0.33	2.40	1.48	1.31	0.17	1.23	0.34	2.21
DHS	Zn	5.82	1.41	2.68	1.60	1.28	0.39	2.08	1.14	1.34	0.62	1.56	0.76	2.32
LWS	Zn	3.09	1.76	4.23	2.62	4.70	0.99	4.35	0.65	1.52	0.42	2.48	0.68	3.38
Season pooled (Zn)		4.32		2.64		2.62		3.06		1.40		1.68		

Abbreviations: DCS, dry cold season; DHS, dry hot season; KANSDS, Kansanshi downstream; KISDS, Kisengisengi downstream; KISGS, Kisengisengi source; KYABR, Kyansafwa bridge; KYAFUS, Kyafukuma upstream; LWS, long wet season; SD, standard deviation; SMWPO, Smelter waste‐pond outlet.

**TABLE A7:** Translocation factors (TF) by *Typha domingensis* for As, Cu, Pb and Zn in the streams of Kansanshi sub‐catchment on the north‐western Zambian Copperbelt.

Season	Metal	KISGS	± SD	KISDS	± SD	KYABR	± SD	KYAFUS	± SD	SMWPO	± SD	KANSDS	± SD	Site pooled
DCS	As	0.49	0.37	1.78	1.18	2.22	1.81	3.39	1.04	0.87	0.21	0.30	0.07	1.505
DHS	As	0.59	0.35	1.22	1.19	0.76	0.26	1.64	1.28	0.87	0.47	0.62	0.35	0.945
LWS	As	0.68	0.37	1.41	0.94	1.40	1.08	1.62	1.67	0.96	0.59	0.91	0.51	1.166
Season pooled (As)		0.60		1.47		1.55		2.08		0.91		0.61		
DCS	Cu	0.51	0.14	0.80	0.30	0.37	0.14	0.25	0.21	0.29	0.27	1.07	0.18	0.526
DHS	Cu	0.52	0.41	1.00	0.00	0.64	0.38	0.42	0.30	0.24	0.21	0.61	0.33	0.571
LWS	Cu	0.80	0.36	0.63	0.47	0.85	0.29	0.25	0.09	0.19	0.12	0.61	0.38	0.532
Season pooled (Cu)		0.63		0.80		0.62		0.30		0.23		0.74		
DCS	Pb	0.60	0.32	1.38	1.07	1.90	0.62	1.65	0.52	1.09	0.26	0.59	0.20	1.220
DHS	Pb	0.90	0.30	1.13	0.50	1.03	0.43	1.63	0.63	0.83	0.17	1.07	0.27	1.091
LWS	Pb	0.78	0.21	1.81	0.88	1.13	0.28	1.29	0.68	1.04	0.24	0.97	0.20	1.169
Season pooled (Pb)		0.76		1.45		1.39		1.49		0.99		0.90		
DCS	Zn	0.50	0.17	1.10	0.95	1.34	0.76	2.71	1.38	0.41	0.32	0.56	0.40	1.071
DHS	Zn	0.94	0.59	1.20	0.55	1.01	0.40	2.19	1.40	0.74	0.11	1.22	0.55	1.209
LWS	Zn	0.69	0.22	1.40	0.36	1.36	0.55	1.47	1.26	0.82	0.16	1.13	0.50	1.127
Season pooled (Zn)		0.71		1.24		1.27		2.00		0.67		1.01		

Abbreviations: DCS, dry cold season; DHS, dry hot season; KANSDS, Kansanshi downstream; KISDS, Kisengisengi downstream; KISGS, Kisengisengi source; KYABR, yansafwa bridge; KYAFUS, Kyafukuma upstream; LWS, long wet season; SD, standard deviation; SMWPO, Smelter waste‐pond outlet.

**TABLE A8:** Joint interaction effect size values ($\eta_{p}^{2}$) from two‐factor MANOVA of As, Cu, Pb and Zn in sediment and *Typha domingensis* . Also included are the BAF and TF partial Eta‐squared values from the joint contribution the metals on the phytopotential of *T. domingensis* in streams of Kansanshi sub‐catchment, north‐western Zambian Copperbelt.

Factor	Partial Eta^2^ ($\eta_{p}^{2}$)	95% CI^a^
Sediment		
Site	0.58	[0.55, 1.00]
Season	0.13	[0.08, 1.00]
Site × Season	0.15	[0.10, 1.00]
*Typha domingensis*		
Site	0.41	[0.38, 1.00]
Season	0.16	[0.13, 1.00]
Site × Season	0.13	[0.10, 1.00]
Bioaccumulation factor		
Site	0.53	[0.50, 1.00]
Season	0.23	[0.17, 1.00]
Site × Season	0.23	[0.07, 1.00]
Translocation factor		
Site	0.37	[0.33, 1.00]
Season	0.06	[0.02, 1.00]
Site × Season	0.13	[0.08, 1.00]

^a^ The 95% confidence intervals (CI) are one‐sided with the upper bound fixed at 1.00.
